# Diagnostic Accuracy of the Social Attention and Communication Surveillance–Revised With Preschool Tool for Early Autism Detection in Very Young Children

**DOI:** 10.1001/jamanetworkopen.2021.46415

**Published:** 2022-03-11

**Authors:** Josephine Barbaro, Nancy Sadka, Melissa Gilbert, Erin Beattie, Xia Li, Lael Ridgway, Lauren P. Lawson, Cheryl Dissanayake

**Affiliations:** 1 Olga Tennison Autism Research Centre, School of Psychology and Public Health, La Trobe University, Melbourne, Australia; 2 Cooperative Research Centre for Living with Autism (Autism CRC), The University of Queensland, Indooroopilly, Queensland, Australia; 3 Department of Mathematics and Statistics, La Trobe University, Melbourne, Australia; 4 Judith Lumley Centre, La Trobe University, Melbourne, Australia

## Abstract

**Importance:**

Early identification of children on the autism spectrum is crucial to facilitate access to early supports and services for children and families. The need for improved early autism identification tools is highlighted by the lack of sufficient diagnostic accuracy in current tools.

**Objectives:**

To examine the diagnostic accuracy of the Social Attention and Communication Surveillance–Revised (SACS-R) and SACS-Preschool (SACS-PR) tools when used with a large, community-based, convenience sample and identify the prevalence of autism in this sample.

**Design, Setting, and Participants:**

This diagnostic accuracy study was conducted in Melbourne, Australia, training maternal and child health nurses who monitored 13 511 children aged 11 to 42 months using the SACS-R and SACS-PR during their routine consultations (June 1, 2013, to July 31, 2018). Children identified as being at high likelihood for autism (12-24 months of age: n = 327; 42 months of age: n = 168) and at low likelihood for autism plus concerns (42 months of age: n = 28) were referred by their maternal and child health nurse for diagnostic assessment by the study team. Data analysis was performed from April 13, 2020, to November 29, 2021.

**Exposures:**

Children were monitored with SACS-R and SACS-PR at 12, 18, 24, and 42 months of age.

**Main Outcomes and Measures:**

Diagnostic accuracy of the SACS-R and SACS-PR was determined by comparing children’s likelihood for autism with their diagnostic outcome using clinical judgment based on standard autism assessments (Autism Diagnostic Observation Schedule–Second Edition and Autism Diagnostic Interview–Revised).

**Results:**

A total of 13 511 children (female: 6494 [48.1%]; male: 7017 [51.9%]) were monitored at least once with the SACS-R at their 12-, 18-, and 24-month-old routine maternal and child health consultations (mean [SD] age, 12.3 [0.59] months at 12 months; 18.3 [0.74] months at 18 months; 24.6 [1.12] months at 24 months) and followed up at their 42-month maternal and child health consultation (mean [SD] age, 44.0 [2.74] months) with SACS-PR (8419 [62.3%]). At 12 to 24 months, SACS-R showed high diagnostic accuracy, with 83% positive predictive value (95% CI, 0.77-0.87) and 99% estimated negative predictive value (95% CI, 0.01-0.02). Specificity (99.6% [95% CI, 0.99-1.00]) was high, with modest sensitivity (62% [95% CI, 0.57-0.66]). When the SACS-PR 42-month assessment was added, estimated sensitivity increased to 96% (95% CI, 0.94-0.98). Autism prevalence was 2.0% (1 in 50) between 11 and 30 months of age and 3.3% (1 in 31) between 11 and 42 months of age.

**Conclusions and Relevance:**

The SACS-R with SACS-PR (SACS-R+PR) had high diagnostic accuracy for the identification of autism in a community-based sample of infants, toddlers, and preschoolers, indicating the utility of early autism developmental surveillance from infancy to the preschool period rather than 1-time screening. Its greater accuracy compared with psychometrics of commonly used autism screening tools when used in community-based samples suggests that the SACS-R+PR can be used universally for the early identification of autism.

## Key Points

**Question:**

Can a developmental surveillance approach be used to train professionals to accurately identify infants, toddlers, and preschoolers on the autism spectrum?

**Findings:**

In this diagnostic accuracy study including 13 511 children aged 11 to 42 months, maternal and child health nurses were trained to use the Social Attention and Communication Surveillance–Revised (SACS-R) and SACS-Preschool (SACS-PR) tools during well-child checkups at 11 to 30 months of age and at follow-up (at 42 months of age). Those children identified as being at high likelihood for autism underwent diagnostic assessments; results indicated the SACS-R with SACS-PR (SACS-R+PR) had very high diagnostic accuracy for early autism detection.

**Meaning:**

Results of this study suggest that the SACS-R+PR population-based developmental surveillance program may be used universally for the early identification of autism.

## Teaser

This diagnostic study assesses the accuracy of the Social Attention and Communication Surveillance–Revised and Social Attention and Communication Surveillance–Preschool in identifying children aged 11 to 42 months on the autism spectrum.

## Introduction

Approximately 2% of individuals worldwide are on the autism spectrum,^1,2^ with some studies reporting a prevalence of 4% or higher.^3^ Early identification is crucial for children on the autism spectrum and their families because it facilitates early diagnosis, and access to supports and services, which greatly improves outcomes^4,5,6,7^; however, the mean age of diagnosis in childhood is still approximately 4 years.^8,9^ Early identification can be achieved via single-time autism screening in the general population (level 1 tools) or targeted groups (level 2 tools), such as siblings of children on the autism spectrum or those in clinical settings (eg, hospitals). Other approaches include developmental surveillance during which children are repeatedly monitored over time.^10,11^ The information source can also vary, with tools using parental or caregiver report, professional observations, or a combination of both.^12,13^

Many early autism screening tools exhibit limited accuracy and sensitivity and, in some cases, limited reporting of sufficient psychometrics to determine overall diagnostic accuracy, particularly in community-based samples.^13,14^ A recent systematic review^14^ noted that few level 1 and 2 autism screeners had undergone multiple validation studies that reported positive predictive values (PPVs) and negative predictive values (NPVs), with several studies reporting neither value. In a systematic review^15^ of universal autism screening in primary care, including the Infant-Toddler Checklist (ITC)^16^ and the Modified Checklist for Autism in Toddlers (M-CHAT) and its iterations,^17,18,19^ the authors noted that few studies included sufficient participant numbers to establish population sensitivity, specificity, and PPV. In addition, psychometric properties reported were modest and/or wide-ranging, indicating lack of, or inconsistency in, the diagnostic accuracy of these tools.

Given these discrepancies, the utility of universal autism screening has been questioned. The US Preventive Services Task Force cited insufficient evidence to recommend autism-specific screening,^20^ instead recommending routine general developmental surveillance. The American Academy of Pediatrics^21,22^ recommends developmental surveillance between 9 and 30 months and autism-specific screening at 18 and 24 months because of the positive impacts of early supports and services. However, a false dichotomy^23^ has been made between screening and surveillance, with screening using an evidence-based tool and surveillance, in the form of active surveillance, using the routine gathering of information from various sources.^24^ Rather than viewing these as separate processes, it is beneficial and time-efficient to integrate autism-specific screening tools into a developmental surveillance framework, using a validated tool as part of the information-gathering developmental surveillance process. Under this approach, all young children are routinely and repeatedly monitored for the early signs of autism whenever they interact with a health care professional (eg, primary care nurses, physicians, or pediatricians).^12^ Dai et al^25^ reported the incremental utility of screening children for autism at 18 and 24 months to maximize opportunities for early identification. Thus, the use of an autism-specific screening tool within a developmental surveillance framework appears to have utility in earlier identification and diagnosis.

The Social Attention and Communication Study (SACS)^10^ implemented a developmental surveillance framework to identify young children on the autism spectrum. It used prospective routine monitoring of infants and toddlers with brief developmentally appropriate checklists focused on the key markers of autism. A critical difference in this study was the use of a community-based sample rather than a clinical or high-likelihood sibling sample, which may not be representative of the general population of children on the autism spectrum because child outcomes, cognition, and autism prevalence vary by ascertainment strategy and multiplex or simplex status.^26,27,28,29^ In a large-scale study^30^ of 22 168 children aged 8 to 24 months, the SACS tool was used by trained maternal and child health (MCH) nurses who monitored children during routine consultations at 12, 18, and 24 months. Results revealed good to excellent psychometric properties; autism diagnoses at 24 months indicated a PPV of 81%, with all remaining children having language and/or developmental delays. Because the children could not be followed up to identify false-negative results, sensitivity (83.8%) and specificity (99.8%) were estimated using autism prevalence at the time, with NPV not calculated. The Social Attention and Communication Surveillance–Revised (SACS-R) tool was then developed, for which items from each age-based checklist found most predictive of an autism diagnosis at 24 months were designated as key items for referral.^30^ The SACS-Preschool (SACS-PR) was developed as a fourth check as part of this developmental surveillance program and to identify any false-negative results between 12 and 24 months.^31^

The study objectives were to determine diagnostic accuracy of the SACS-R and SACS-PR in a large community-based sample and to report estimated autism prevalence. We hypothesized that the psychometric properties of the SACS-R with the SACS-PR (SACS-R+PR) would be similar to those reported for the SACS and that the prevalence of autism would be similar to that recently reported (approximately 2%).^1,2^

## Methods

Before study commencement, approval was granted from the La Trobe University Human Ethics Committee and the Victorian Department of Education and Early Childhood Development. This diagnostic accuracy study followed the Standards for Reporting of Diagnostic Accuracy (STARD) reporting guidelines for diagnostic accuracy studies.^32,33^

### Participants

#### Phase 1

**Figure 1.**
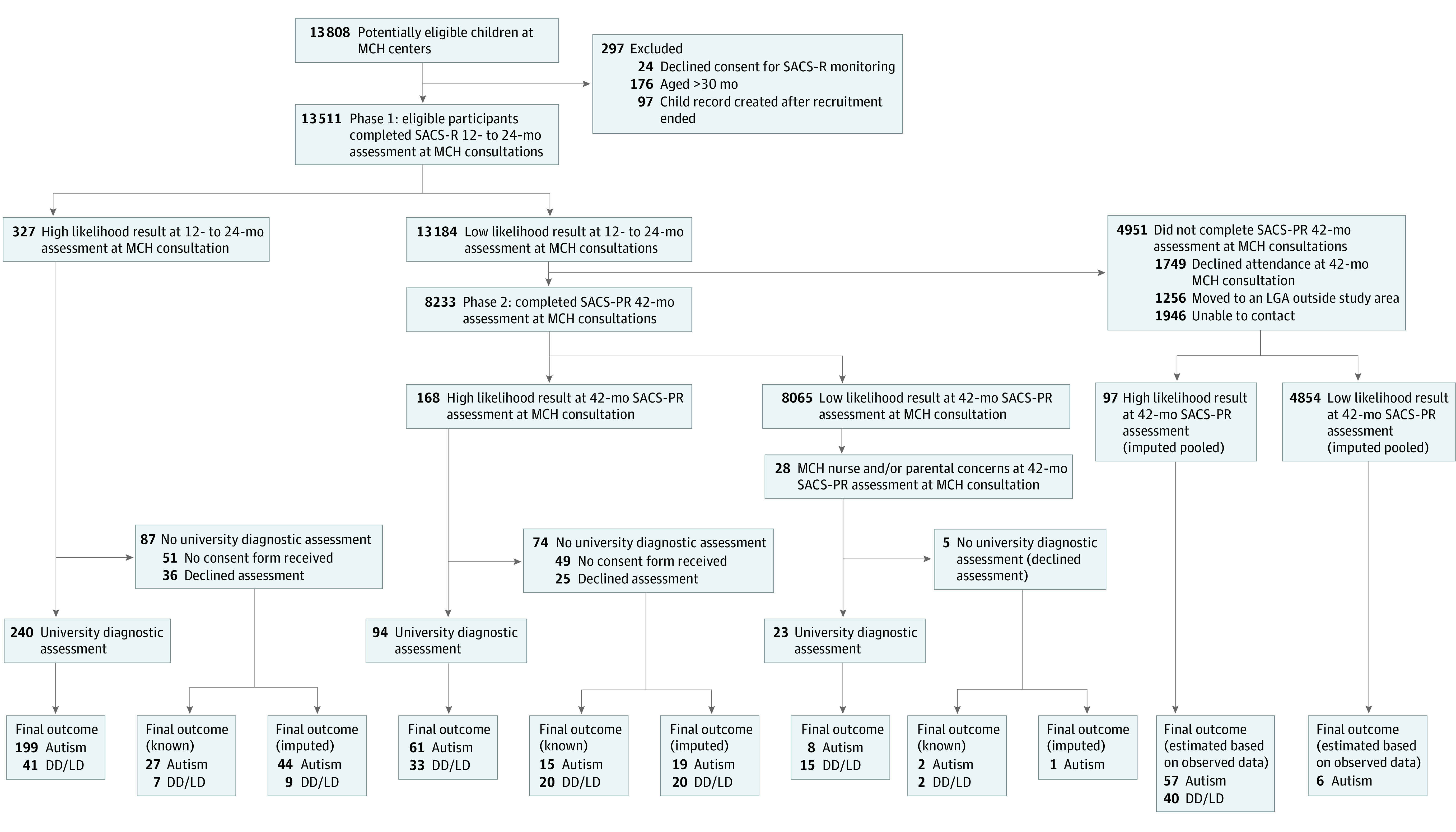
Standards for Reporting of Diagnostic Accuracy Participant Flow Diagram DD/LD indicates developmental delay and/or language delay; LGA, local government area; MCH, maternal and child health; SACS-PR, Social Attention and Communication Surveillance–Preschool; SACS-R, Social Attention and Communication Surveillance–Revised.

Children were eligible to participate if they were within the specified age range (11-30 months) during the recruitment period (June 1, 2013, to March 31, 2015) and attended a routine MCH consultation, available universally to all children living in the State of Victoria, Australia, from birth to 3.5 years of age. The ethics committees granted approval for an opt-out approach. Overall, 13 511 eligible children were prospectively monitored using SACS-R by 126 trained MCH nurses from 8 local government areas between June 1, 2013, and October 31, 2016, with participants forming a convenience sample. Further details are in the eMethods in the Supplement. Of these, 327 (2.4%) were identified as having a high likelihood of autism based on SACS-R and referred for a standard autism diagnostic assessment at La Trobe University, Melbourne, Australia; 240 children (73.4%) underwent this assessment after written parental or caregiver informed consent was received (Figure 1).

#### Phase 2

The sample was followed up at their 42-month MCH consultation (October 1, 2014, to July 31, 2018) using the SACS-PR, with 8419 children (62.3%) assessed, which included children who had already been identified at high likelihood for autism using SACS-R (n = 186) consistent with the 42-month MCH consultation attendance rates across the state (63.2%).^34,35,36^ Again, ethical approval for an opt-out approach was granted. An additional 168 children who had low-likelihood results using the SACS-R but had high-likelihood results using SACS-PR were referred for a university diagnostic assessment; 94 children (56.0%) completed the assessment after written parental or caregiver informed consent was received. Twenty-eight children at low likelihood on the SACS-PR but with MCH nurse and/or parental or caregiver concerns regarding their development were referred for a university diagnostic assessment, with 23 assessed (82.1%) (Figure 1).^32^

### Measures

#### The SACS-R Tool

The SACS-R is an observation-based, early developmental surveillance tool for autism designed for use in community-based samples that comprises 12 to 15 early social-communication behaviors observed at 12, 18, and 24 months (age range, 11-30 months) (eTable 1 in the Supplement for age range of each checklist). Each SACS-R checklist has 5 key items found to be most predictive of a later autism diagnosis.^30^ Before administering the SACS-R, assessors undergo training to determine whether a child is presenting with typical or atypical behavior for each item rather than simply noting behavior as present or absent because behavior can be atypical due to absence, infrequency, or inconsistency, or when not used in combination with other behaviors.^10,11^ Items can be readministered up to 3 times if not initially observed. Parental or caregiver report can be used if required, with the SACS-R training providing guidance on how to ask parents or caregivers to give detailed, explicit examples of the child’s behavior. If required, children can return for reassessment within a short time frame. Children with atypical behavioral presentation on at least 3 key items are considered at high likelihood for autism and referred for further assessment (eTable 2 in the Supplement).

#### The SACS-PR Tool

Based on the SACS-R tool and incorporating other common markers of autism in preschool-aged children, the SACS-PR was designed to accompany the SACS-R for children 31 to 60 months of age.^31^ The SACS-PR has additional repetitive, stereotyped, and sensory behaviors and interests added, given that these behaviors are more evident in preschoolers on the autism spectrum.^31,37,38,39,40^ Assessors determine whether a child’s behavior is typical or atypical across 22 behavioral items, with children assessed as having atypical behavior for at least 3 of the 8 key items considered at high likelihood of autism (eTable 2 in the Supplement). Results indicate that the SACS-PR has an area under the curve of 0.95, indicating excellent goodness of fit in discriminating between preschoolers on the autism spectrum and those with developmental and/or language delays.^31^

### Diagnostic Assessments

Children referred between the ages of 11 and 30 months (phase 1) for a diagnostic assessment were seen by the university team at 6-month intervals until 30 months of age and followed up at 42 months. Standard diagnostic assessments (the reference standard) were conducted by trained, experienced clinicians at La Trobe University. Tools administered included the Autism Diagnostic Observation Schedule–Second Edition (ADOS-2; modules 1-3) or the ADOS-Toddler (ADOS-T),^37,38^ Mullen Scales of Early Learning,^39^ and a brief developmental interview *or* the Autism Diagnostic Interview-Revised (ADI-R),^40^ dependent on the child’s age and diagnostic status.^41^ eTable 3 in the Supplement provides details of all tools. Based on clinical judgment and information gained from formal assessments, observations, developmental history, and previous assessments by other health professionals, 2 clinicians determined a diagnostic outcome for all children by 24 months and confirmed at 42 months (ie, autism or developmental and/or language delay). Further details regarding the tools, assessment procedure, clinician training, research reliability, diagnostic criteria and stability, are included in eMethods in the Supplement.

### Statistical Analysis

Statistical analyses were conducted using SPSS Statistics for Windows, version 26.0 (IBM Corp). Data analysis was performed from April 13, 2020, to November 29, 2021. With assumptions tested prior to analyses, independent samples *t* tests and Mann-Whitney U tests were used to identify differences in mean age, ADOS-2, ADI-R, and verbal, nonverbal, and overall developmental quotient using Mullen Scales of Early Learning scores between groups (all tests were 2-sided with α = .05). Sex ratios were calculated overall and for each age group (12, 18, 24, and 42 months). To examine the diagnostic accuracy, PPV was calculated using observed diagnostic outcomes; true sensitivity, specificity, and NPV could not be calculated because of imputed values needed for missing diagnostic outcome data, unavoidable in large-scale, community-based studies. These values were estimated using observed or known and imputed data. For children who were referred but did not complete a university diagnostic assessment, diagnostic outcomes obtained from parents or caregivers, MCH nurses, and/or other professionals (eg, pediatricians) were used where known, with multiple imputation used to impute unknown diagnostic outcomes. For children who did not attend their 42-month follow-up MCH consultation, multiple imputation was used to determine outcomes of high or low likelihood; estimates of autism and developmental and/or language delay were used based on known diagnostic outcomes. Further details on assumption testing, analysis plan, missing data, multiple imputation models, and analyses are available in the eMethods in the Supplement.

## Results

### Sample Characteristics

In phase 1, 13 511 children were monitored using the SACS-R at least once (female: 6494 [48.1%]; male: 7017 [51.9%]) at their 12-, 18-, and 24-month routine MCH consultations (mean [SD] age, 12.3 [0.59] months at 12 months; 18.3 [0.74] months at 18 months; 24.6 [1.12] months at 24 months) and followed up in phase 2 with the SACS-PR (8419 [62.3%]) at their 42 month MCH consultation (44.0 [2.74] months); ethnicity data were not available for children not assessed by the university team.

Overall, 31 708 SACS-R and SACS-PR assessments were completed during the study. A total of 430 of the 523 children (82.2%) referred to the study team completed a university assessment or had a known diagnostic outcome provided. There were 4971 children (37.6%) who were not followed up using the SACS-PR because of families moving to a nonparticipating local government area (n = 1256), not attending their 42-month MCH appointment (n = 1749), or not being able to be contacted by their MCH nurse (n = 1946). Figure 1 details the STARD^32^ study flowchart. eFigure 1 in the Supplement includes further details.

**Figure 2.**
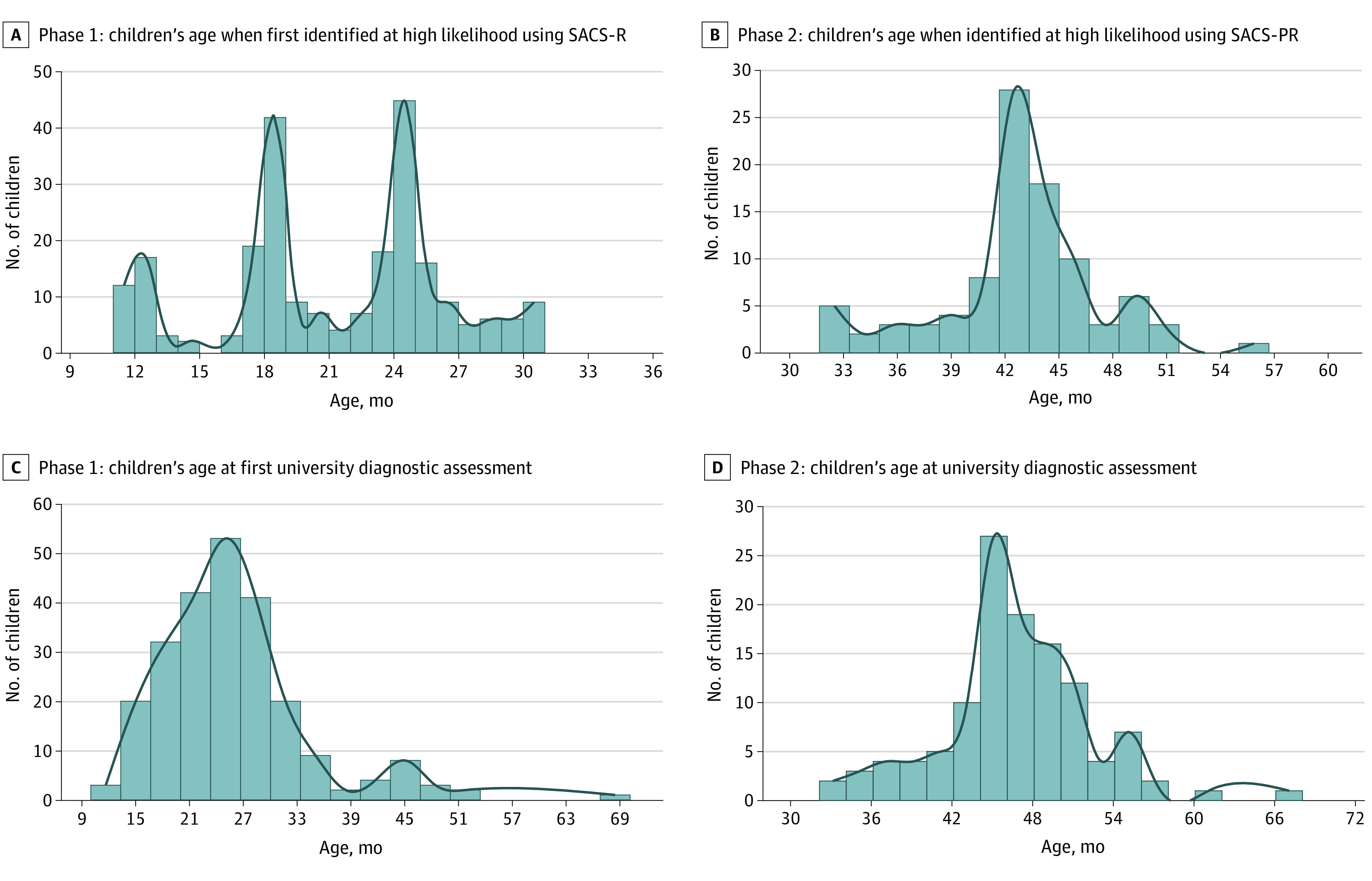
Distribution of Children’s Ages When First Identified at High Likelihood for Autism Using SACS-R and at Phase 1 and 2 University Diagnostic Assessment SACS-PR indicates SACS-Preschool; SACS-R, SACS-Revised.

eFigure 2 in the Supplement displays the number of children monitored within each age group during phase 1; Figure 2A shows the distribution of children’s ages when first identified as being at high likelihood for autism. Mean (SD) age when first identified as being at high likelihood on SACS-R was 21.2 (5.1) months. Figure 2B shows the distribution of children’s ages when identified as being at high likelihood using SACS-PR, with a mean (SD) age of 42.8 (4.4) months.

Table 1 presents child and family demographic characteristics for the 357 children who underwent at least 1 university diagnostic assessment. Parents and caregivers completed a demographic questionnaire, which included questions regarding parental country of birth and ethnicity, to enable comparison of the sample with the population of greater Melbourne. Ethnicity options were defined by the parent or caregiver completing the questionnaire and were later grouped by the researchers for analyses. No statistically significant differences were found between the autism and the developmental delay and/or language delay groups for any demographic characteristics. Median family income ranged from US $90 155 to US $109 135, and 254 families (71.1%) spoke only English at home; most mothers (n = 246 [68.9%]) and fathers (n = 230 [64.4%]) were White and born in Australia (mothers: n = 238 [66.7%]; fathers: n = 215 [60.2%]).^4^ Parents were more highly educated compared with greater Melbourne, with 202 mothers (56.6%) and 153 fathers (42.9%) university educated.^42^ A summary of clinical assessment scores by age groups and final diagnostic status and comparisons of the developmental variables are presented in eTable 4 in the Supplement with further details in the eResults.

**Table 1.** Child and Family Characteristics at Last Diagnostic Assessment

Characteristic	No. (%) of children		
	Autism (n = 268)	DD and/or LD (n = 89)	Total (N = 357)
Sex			
Male	208 (77.6)	71 (79.8)	279 (78.2)
Female	60 (22.4)	18 (20.2)	78 (21.8)
Mother’s country of birth (highest responses)			
Australia	183 (68.3)	55 (61.8)	238 (66.7)
India	8 (3.0)	3 (3.4)	11 (3.1)
Sri Lanka	9 (3.4)	0	9 (2.5)
New Zealand	5 (1.9)	3 (3.4)	8 (2.2)
Philippines	7 (2.6)	1 (1.1)	8 (2.2)
United Kingdom	6 (2.2)	2 (2.2)	8 (2.2)
Father’s country of birth (highest responses)			
Australia	158 (59.0)	57 (64.0)	215 (60.2)
India	9 (3.4)	3 (3.4)	12 (3.4)
United Kingdom	5 (1.9)	6 (6.8)	11 (3.1)
New Zealand	7 (2.6)	3 (3.4)	10 (2.8)
Sri Lanka	8 (3.0)	0	8 (2.3)
Mother’s race and ethnicity^a^			
African	4 (1.5)	2 (2.2)	6 (1.7)
Asian or Middle Eastern	59 (22.0)	15 (16.9)	74 (20.7)
Indigenous	3 (1.1)	1 (1.1)	4 (1.1)
Mixed background	4 (1.5)	2 (2.2)	6 (1.7)
White	183 (68.3)	63 (70.8)	246 (68.9)
Unspecified or not reported	8 (3.0)	0	8 (2.2)
Father’s race and ethnicity^a^			
African	4 (1.5)	4 (4.5)	8 (2.2)
Asian or Middle Eastern	54 (20.1)	10 (11.2)	64 (17.9)
Indigenous	2 (0.7)	1 (1.1)	3 (0.8)
Mixed background	3 (1.1)	2 (2.2)	5 (1.4)
White	168 (62.7)	62 (69.7)	230 (64.4)
Unspecified or not reported	30 (11.2)	4 (4.5)	34 (9.5)
Mother’s highest educational level completed			
Primary	1 (0.4)	1 (1.1)	2 (0.6)
Some secondary	25 (9.3)	3 (3.4)	28 (7.8)
Secondary	16 (6.0)	11 (12.4)	27 (7.6)
TAFE	44 (16.4)	11 (12.4)	55 (15.4)
Trade certificate	20 (7.5)	3 (3.4)	23 (6.4)
University	151 (56.3)	51 (57.3)	202 (56.6)
Not reported	3 (1.1)	3 (3.4)	3 (1.7)
Father’s highest educational level completed			
Primary	2 (0.7)	1 (1.1)	3 (0.8)
Some secondary	29 (10.8)	8 (9.0)	37 (10.4)
Secondary	26 (9.7)	10 (11.2)	36 (10.1)
TAFE	17 (6.3)	8 (9.0)	25 (7.0)
Trade certificate	41 (15.3)	16 (18.0)	57 (16.0)
University	115 (42.9)	38 (42.7)	153 (42.9)
Not reported	24 (9.0)	1 (1.1)	25 (7.0)
Annual family income, US $			
<33 214	28 (10.4)	4 (4.5)	32 (9.0)
33 216-52 195	19 (7.1)	6 (6.7)	25 (7.0)
52 196-71 175	38 (14.2)	5 (5.6)	43 (12.0)
71 176-90 155	32 (11.9)	8 (9.0)	40 (11.2)
90 156-109 135	23 (8.6)	10 (11.2)	33 (9.2)
109 136-128 115	19 (7.1)	11 (12.4)	30 (8.4)
128 116-147 095	8 (3.0)	7 (7.9)	15 (4.2)
147 096-166 075	10 (3.7)	1 (1.1)	11 (3.1)
>166 075	26 (9.7)	11 (12.4)	37 (10.4)
Do not wish to answer or not reported	58 (21.6)	20 (22.5)	78 (21.8)
Language(s) spoken at home			
English only	187 (69.8)	67 (75.3)	254 (71.1)
≥2 Languages (including English)	63 (23.5)	13 (14.6)	76 (21.3)
Another language only (no English spoken)	9 (3.4)	3 (3.4)	12 (3.4)
Not reported	2 (0.7)	0	2 (0.6)

Abbreviations: DD, developmental delay; LD, language delay; TAFE, technical and further education.

^a^ Parents and caregivers completed a demographic questionnaire, which included questions regarding parental country of birth and ethnicity, to enable comparison of the sample with the population of greater Melbourne. Ethnicity options were defined by the parent or caregiver completing the questionnaire and were later grouped by the researchers for analyses.

eTable 5 in the Supplement displays the breakdown of children’s university diagnostic assessments; Figure 2C and D shows the distribution of children’s ages at their first university diagnostic assessment. Mean (SD) ages were 14.3 (0.8) months at the 12-month diagnostic assessment, 19.8 (1.2) months at the 18-month diagnostic assessment, 26.4 (2.0) months at the 24-month diagnostic assessment, and 46.6 (5.5) months at the 42-month diagnostic assessment (eTable 4 in the Supplement).

### Diagnostic Accuracy

#### Phase 1

A total of 327 children (2.4%) of the 13 511 monitored using SACS-R were identified as having a high likelihood of autism (62 at 12 months, 107 at 18 months, and 158 at 24 months) (Figure 1). Of the 240 children who completed a university assessment, 199 (82.9%) met autism spectrum disorder (ASD) diagnostic criteria; the remaining 41 children (17.1%) had developmental delay and/or language delay. Known diagnostic outcomes and imputation resulted in 71 children added to the autism group, totaling 270.

#### Phase 2

A total of 168 children were identified as being at high likelihood on the SACS-PR at 42 months. Sixty-one of the 94 children (64.9%) assessed by the university team met ASD diagnostic criteria; the remaining 33 (35.1%) had developmental delay and/or language delay. Twenty-eight children with a low likelihood result on the SACS-PR but referred because of parental and/or MCH nurse concerns were assessed at the university; 8 (28.5%) met diagnostic criteria for ASD. Of the 5 children who did not have a university assessment, 2 had known diagnostic outcomes, and 1 child’s diagnostic outcome was imputed as autism. A total of 4951 children did not complete a SACS-PR assessment because of nonattendance at their 42-month MCH consultation. Multiple imputation was used to estimate that 63 children on the autism spectrum would have been in this group. No typically developing children were identified as being at high likelihood on the SACS-R or SACS-PR.

eTables 6 and 7 in the Supplement contain cross-tabulations of the SACS-R (12-24 months) results and SACS-R+PR (12-42 months), with the reference standard (diagnostic outcome). Figure 1 includes further details of participant flow. Calculation of diagnostic accuracy based on observed and imputed data is detailed in Table 2, and PPV on observed data only is in Table 3. The eMethods in the Supplement present definitions and formulas. The SACS-R had a PPV of 83% (95% CI, 0.77-0.87) (based on observed data); the estimated NPV was 99% (95% CI, 0.02-0.01), sensitivity was 62% (95% CI, 0.57-0.66), and specificity was 99.6% (95% CI, 0.99-1.00) (based on observed and imputed data). The SACS-R+PR had a PPV of 78% (95% CI, 0.73-0.82) (based on observed data); the estimated NPV was 99.9% (95% CI, 0.002-0.001), sensitivity was 96% (95% CI, 0.94-0.98), and specificity was 98% (95% CI, 0.98-0.99) (based on observed and imputed data). For individual checklists (based on observed data), the SACS-R+PR had a PPV of 74% at 12 months, 82% at 18 months, 86% at 24 months, and 65% at 42 months.^43^

**Table 2.** Diagnostic Accuracy Analysis for SACS-R Results Between 12 and 24 Months and SACS-R+PR Results Between 12 and 42 Months

Measure	Value (95% CI)
SACS-R (12-24 mo)	
Study prevalence	0.03 (0.03-0.04)
Estimated sensitivity	0.62 (0.57-0.66)
Estimated specificity	1.00 (0.99-1.00)
Predictive values	
Actual PPV	0.83 (0.77-0.87)
Estimated 1 − NPV	0.01 (0.02-0.01)
SACS-R+PR (12-42 mo)	
Study prevalence	0.03 (0.03-0.04)
Estimated sensitivity	0.96 (0.94-0.98)
Estimated specificity	0.99 (0.98-0.99)
Predictive values	
Actual PPV	0.78 (0.73-0.82)
Estimated 1 − NPV	0.001 (0.002-0.001)

Abbreviations: NPV, negative predictive value; PPV, positive predictive value; SACS-R, Social Attention and Communication Surveillance–Revised; SACS-R+PR, Social Attention and Communication Surveillance–Revised combined with Preschool.

**Table 3.** SACS-R, SACS-PR, and SACS-R+PR Positive Predictive Values Based on Observed Diagnostic Outcomes at Each Age Group

Outcome	Age group, mo					
	SACS-R				SACS-PR	SACS-R+PR
	12	18	24	12-24	42	12-42
No. of children at high likelihood for autism	35	84	121	240	94	334
No. of children on the autism spectrum	26	69	104	199	61	260
No. of children with DD and/or LD	9	15	17	41	33	74
PPV for autism, %	74.3	82.1	86.0	82.9	64.9	77.8

Abbreviations: DD, development delay; LD, language delay; PPV, positive predictive value; SACS-PR, Social Attention and Communication Surveillance–Preschool; SACS-R, Social Attention and Communication Surveillance–Revised; SACS-R+PR, Social Attention and Communication Surveillance–Revised combined with Preschool.

### Prevalence

Autism prevalence between 11 and 30 months of age was 2.0% (1 in 50), with 270 of the 13 511 children monitored assigned to the autism group (diagnosed or imputed). When incorporating children on the autism spectrum identified at 42 months, this number increased to 3.3% (1 in 31), with 439 in the autism group (diagnosed or imputed).

## Discussion

Children on the autism spectrum can be reliably diagnosed in the second year of life.^44^ However, no screening tool has been recommended for universal use because previous research^14,15^ indicates commonly used screening tools have low diagnostic accuracy when used in community-based samples. Early identification tools with high diagnostic accuracy are critical, given the known positive impacts of early diagnosis, supports and services for children and their families.^4,5,6,7^

### Diagnostic Accuracy

The key objective in this study was to implement the SACS-R and SACS-PR developmental surveillance tools in a large, community-based sample of very young children to determine diagnostic accuracy. The SACS-R+PR has high diagnostic accuracy in prospectively identifying community-based infants, toddlers, and preschoolers on the autism spectrum, replicating the results of the original SACS while also reporting more robust indexes of diagnostic accuracy. Furthermore, *no* children identified as being at high likelihood by the SACS-R or SACS-PR were typically developing; thus, all children required supports and services. The SACS-R had excellent PPV (82.6%), NPV (98.7%), and specificity (99.6%) and moderate sensitivity (61.5%) when used between 12 and 24 months of age; however, when the SACS-PR was added at 42 months, sensitivity increased to 96.1%, highlighting the critical importance of a developmental surveillance model to identify false-negative results at each subsequent consultation. Thus, SACS-R+PR psychometrics are well above ideal minimum psychometric properties (≥70%).^13,43^ Combined, these data provide evidence of the high diagnostic accuracy of the SACS-R+PR between 12 and 42 months of age.^45^

These findings indicate that the SACS-R+PR has better psychometrics than other commonly used early autism screening tools, such as the ITC, Brief Infant-Toddler Social and Emotional Assessment (BITSEA), and M-CHAT, with most studies not using purely community-based samples.^46^ A previous study^47^ of children with and without a family history of autism found that the ITC has moderate overall psychometrics in distinguishing autism and nonautism; sensitivity ranged from 55% to 77%, specificity from 42% to 85%, PPV from 20% to 55%, and NPV from 83% to 94%. A study on the BITSEA in younger siblings of children on the autism spectrum and children with no family history of autism indicated it cannot distinguish between these groups.^48^ In addition, the BITSEA’s 4 autism-specific scales had high specificity (84%-90%) but low sensitivity (40%-52%).^49^ Despite its common use, a meta-analysis^50^ indicated the M-CHAT has a low pooled specificity of 51% and a sensitivity of 83% in children with *previously* identified developmental concerns (ie, not community based). Pooled PPV was also very low (6%) when used in a community-based population without previous developmental concerns. M-CHAT’s NPV could not be established because the samples were not followed up.^46,47,48,49,50^

### Autism Prevalence

A secondary objective was to establish the prevalence of autism in this community-based sample. Prevalence was 1 in 50 (2.0%) between 11 and 30 months of age, consistent with previously reported rates.^1,2^ This prevalence increased to 1 in 31 (3.3%) when children on the autism spectrum were identified at their 42-month consultation. This latter prevalence rate is higher than the latest rates of 2.3% from the Centers for Disease Control and Prevention^2^ and 1.5% to 2.5% from a longitudinal Australian study^51^ of children younger than 7 years.

### Strengths and Limitations

This study has several strengths. Given the debate regarding recommendations for autism screening, with the lack of resolution in part attributable to minimal community-level data,^23^ the current study has provided further large-scale, community-level data to support the use of autism-specific tools during developmental surveillance for autism. As Dai et al^25^ state, there is incremental utility in monitoring children across time following an initial low likelihood result. Had the SACS-R not incorporated multiple checklists across the second year of life, with a SACS-PR follow-up at 42 months, 184 children on the autism spectrum would not have been identified (with 110 receiving a low likelihood result at 12 months, 49 at 18 months, and 25 at 24 months). This finding highlights the importance of a developmental surveillance approach using developmentally appropriate autism-specific tools as best practice in the early identification of autism; the current findings demonstrate that a 1-time screening approach would have missed many children on the autism spectrum. The 168 children on the autism spectrum identified at their 42-month checkup indicates the utility of the SACS-PR (used alongside parental and/or caregiver and MCH nurse concerns) together with the SACS-R to increase the sensitivity of the program.

The mean age of identification using SACS-R (21.2 months) is much lower than the current mean age of diagnosis in Australia (49.2 months),^8^ indicating a large gap between when it is possible to identify children on the autism spectrum and eventual diagnoses in the community. The SACS-R could facilitate earlier access to services and in turn reduce the age of autism diagnoses. An additional strength is the use of a community-based sample in children without known developmental concerns, which replicates how developmental surveillance tools are used in real-world settings, such as primary care.

This study also has some limitations. Not all children underwent a university diagnostic assessment, requiring some diagnostic outcomes to be determined from other sources, the use of multiple imputation, and the estimation of sensitivity, specificity, and NPV. Although preferable that all children received such standard assessments, loss to follow-up is unavoidable in large-scale, community-based research. This unavoidable loss to follow-up required the use of multiple imputation and the estimation of sensitivity, specificity, and NPV. It is also possible that children who were rated as being at low likelihood of autism on the SACS-R and SACS-PR, as well as having no concerns by parents or caregivers or MCH nurses at 42 months, may have a later diagnosis of autism. Given that the study was only able to follow up children to their 42-month checkup, we are unable to report this as part of the current study. However, because the prevalence of autism reported here (3.3%) is higher than that reported in other studies (approximately 2%),^1,2^ the number of missed children is likely very small.

Another potential limitation is the time and resources required for a trained professional to administer the SACS-R and SACS-PR. Although these tools have the benefit of increased diagnostic accuracy because of this requirement, there may be instances in which this could be impractical. Parent- and caregiver-completed tools can offer advantages for health care professionals but with the trade-off of lower psychometrics in part because of parents and caregivers having less knowledge regarding early social-communication development.^12,52^ A parent- or caregiver-led app based on the SACS-R, ASDetect,^53^ may help to overcome this barrier because it includes video examples of typical and atypical social-communication development that parents and caregivers watch before answering the questions.^12,52^ Future research should consider combining tools administered by parents or caregivers and health care professionals.

Although this study investigated the use of the SACS-R and SACS-PR in a discrete setting, the SACS-R has successfully been used in other national and international settings (eg, Female Community Health Volunteers in Nepal,^54^ public health nurses in Japan,^55,56^ community health medical practitioners in China,^57^ and primary care physicians and early childhood educators in Australia),^58,59^ indicating the SACS-R's utility in diverse settings.^57^ The SACS-R has also been directly compared with another tool; Mozolic-Staunton et al^59^ studied administration of the SACS-R and the Parents Evaluation of Developmental Status (PEDS Path ASD)^60,61,62^ in community health and early education settings. The SACS-R had high sensitivity (82.0%) and specificity (99.8%), whereas the PEDS Path ASD had high specificity (99.0%) but low sensitivity (6.7%), indicating that the PEDS Path ASD was more likely than the SACS-R to miss children on the autism spectrum. Future research should consider directly comparing the SACS-R and SACS-PR with other autism screening tools.

## Conclusions

The findings from this large-scale, community-based, diagnostic accuracy study indicate that the SACS-R combined with the SACS-PR is highly accurate in identifying infants, toddlers, and preschoolers on the autism spectrum. This study provides strong evidence supporting the use of the SACS-R+PR as a population-based, developmental surveillance program for the early identification of autism within the general population.

## Notes

Supplement. **eMethods.** Supplementary Methods **eResults.** Supplementary Results **eFigure 1.** Detailed STARD Study Flow Diagram **eTable 1.** Age Ranges for the SACS-R and SACS-PR Checklists **eTable 2.** Behaviors Monitored by the SACS-R at 12-, 18-, and 24-Months, and SACS-PR at 42-Months, Including ‘Key’ Items **eTable 3.** Tools Administered During Diagnostic Assessments Undertaken at Each Age Group **eFigure 2.** Phase 1 Cohort Recruitment and Monitoring by Age Group **eTable 4.** Clinical Assessment Scores for All University Assessments, Grouped by Age at Assessment and Outcome **eTable 5.** Children Who Underwent Diagnostic Assessments One or More Times **eTable 6.** Results of Analysis for SACS-R Between 12-24 Months **eTable 7.** Results of Analysis for SACS-R+PR Between 12-42 Months
eReferences
