# Errors in Conflict of Interest Disclosures

**DOI:** 10.1001/jamanetworkopen.2022.11193

**Published:** 2022-04-13

In the Original Investigation titled “Diagnostic Accuracy of the Social Attention and Communication Surveillance–Revised With Preschool Tool for Early Autism Detection in Very Young Children,”^1^ published March 11, 2022, there were errors in the Conflict of Interest Disclosures. The disclosure statement for Dr Barbaro should have read “Dr Barbaro reported that the Social Attention and Communication Surveillance (SACS) intellectual property has been licensed to the Victorian government, earning royalties for the university. The university also receives funds from individuals signing up for online SACS training. Funds are partially distributed to Dr Barbaro for the background intellectual property.” In addition, the disclosure for Ms Ridgway should have been removed. This article has been corrected.^1^
