# Supplementary material for: Diagnostic Accuracy of the Social Attention and Communication Surveillance–Revised With Preschool Tool for Early Autism Detection in Very Young Children
Source: JAMA Netw Open. 2022 Mar 11;5(3):e2146415. doi: 10.1001/jamanetworkopen.2021.46415 (PMC8917423; doi:10.1001/jamanetworkopen.2021.46415)
Supplement: Supplement. — eMethods. Supplementary Methods eResults. Supplementary Results eFigure 1. Detailed STARD Study Flow Diagram eTable 1. Age Ranges for the SACS-R and SACS-PR Checklists eTable 2. Behaviors Monitored by the SACS-R at 12-, 18-, and 24-Months, and SACS-PR at 42-Months, Including ‘Key’ Items eTable 3. Tools Administered During Diagnostic Assessments Undertaken at Each Age Group eFigure 2. Phase 1 Cohort Recruitment and Monitoring by Age Group eTable 4. Clinical Assessment Scores for All University Assessments, Grouped by Age at Assessment and Outcome eTable 5. Children Who Underwent Diagnostic Assessments One or More Times eTable 6. Results of Analysis for SACS-R Between 12-24 Months eTable 7. Results of Analysis for SACS-R+PR Between 12-42 Months eReferences [file jamanetwopen-e2146415-s001.pdf]

Supplementary Online Content

Barbaro J, Sadka N, Gilbert M, et al. Diagnostic accuracy of the Social Attention and Communication Surveillance–Revised With Preschool tool for early autism detection in very young children. *JAMA Netw Open*. 2022;5(3):e2146415. doi:10.1001/jamanetworkopen.2021.46415

eMethods. Supplementary Methods
eResults. Supplementary Results
eFigure 1. Detailed STARD Study Flow Diagram
eTable 1. Age Ranges for the SACS-R and SACS-PR Checklists
eTable 2. Behaviors Monitored by the SACS-R at 12-, 18-, and 24-Months, and SACS-PR at 42-Months, Including 'Key' Items
eTable 3. Tools Administered During Diagnostic Assessments Undertaken at Each Age Group
eFigure 2. Phase 1 Cohort Recruitment and Monitoring by Age Group
eTable 4. Clinical Assessment Scores for All University Assessments, Grouped by Age at Assessment and Outcome
eTable 5. Children Who Underwent Diagnostic Assessments One or More Times
eTable 6. Results of Analysis for SACS-R Between 12-24 Months
eTable 7. Results of Analysis for SACS-R+PR Between 12-42 Months
eReferences

This supplementary material has been provided by the authors to give readers additional information about their work.

© 2022 Barbaro J et al. *JAMA Network Open.*

eMethods. Supplementary Methods.

Participant Recruitment
A detailed overall study flow diagram is included in eFigure 1.

Maternal and Child Health Nurses, Local Government Areas, and Training Details
The Victorian Maternal and Child Health (MCH) service is free and universally available to children who reside in the state of Victoria, Australia.[1] It provides preventative healthcare for families from birth to school age over a series of 10 routine "key ages and stages" consultations with MCH nurses, with ad hoc consultations also available. Consultations cover issues relating to child development and health, as well as the health and wellbeing of parents/caregivers.[2] MCH centers are located throughout the community, often co-located with other services such as pre-schools. The service has a high uptake with 96% of infants accessing the service at two weeks old. Although reducing slightly over time, service use remains high with almost 68% of children assessed at 24 months of age.[3] MCH nurses in Victoria are required to have current registration with the Australian Health Practitioner Regulation Agency (AHPRA) as a Registered Nurse (Division 1; requiring a 3-year Bachelor of Nursing degree) and Registered Midwife (requiring a 3-year Bachelor of Midwifery degree *or* a graduate diploma/Masters in Midwifery), as well as having an accredited post-graduate degree or diploma in maternal and child health nursing (1-year).[4]

A total of 126 MCH nurses in 88 MCH centers, from eight local government areas (LGAs) in metropolitan Melbourne, Australia participated in the study, following approval from the Municipal Association of Victoria, ethical approval from La Trobe University and the Department of Education and Early Childhood Development, and Informed Consent from each MCH coordinator. The LGAs – Banyule, Bayside, Boroondara, Hume, Kingston, Knox, Moonee Valley, and Nillumbik – were chosen based on their high rates of attendance at their last routine MCH consultation as part of the study (42-months) and proximity to the La Trobe University Melbourne Campus in Bundoora. Proximity to the University of 40km (24.9mi) radius or less was considered important to optimize parents/caregivers' uptake of referrals to the University team for a diagnostic assessment.

A general measure of the socio-economic conditions in areas of Australia from the Australian Bureau of Statistics' Socio-Economic Index for Areas,[5] the 'Index of Relative Socio-Economic Advantage and Disadvantage' (IRSAD), was used to compare the socio-economic conditions of the eight participating LGAs (M = 1058.5, SD = 58.58) with those of the other metropolitan Melbourne LGAs (M = 1025.70, SD = 52.03), indicating no significant differences ($t(29) = 1.489$, $P = .15$).

The Social Attention and Communication Surveillance-Revised (SACS-R) component of practice draws on MCH nurses' knowledge of child developmental assessment, including eliciting parent/caregiver and child responses. The MCH nurses attended one of eight 3½-hour SACS-R training workshops held during a monthly LGA meeting between May and June 2013. The sessions covered the use of the SACS-R tool to monitor children's development for the early signs of autism at the standard 12-, 18-, and 24-month "key ages and stages" MCH consultations. MCH nurses were instructed on how to administer the items and elicit responses on each SACS-R checklist, with the workshop content covering: general autism knowledge; social attention and communication development in infants and toddlers; how to identify whether a child's social communication behaviors are *'typical'* or *'atypical'*, and the early and later signs of autism. Videos were used to illustrate differences in each of the SACS-R behaviors and other signs of autism (e.g., restricted and/or repetitive behaviors), between children on the autism spectrum, children with developmental/language delays, and children with typical development. For further details on the SACS-R training, please see Barbaro et al (2011).[6] Between October 2014 and February 2015, MCH nurses received further training on the administration of the Social Attention and Communication Surveillance-Preschool (SACS-PR) items and referral to the study based on MCH nurse/parental concerns at the 42-month check, during a 2-hour workshop during a monthly LGA meeting.

The MCH nurses were also trained on how to use the *Salesforce* customer relationship management (CRM) platform[7] to enter participant data. The MCH nurse interface of the *Salesforce* platform was designed specifically to meet the needs of this study. All MCH nurses were provided with a SACS-R 'training pack', containing various study materials including brochures, posters, SACS-R manual, Salesforce login cards, participant stickers, participant information and consent forms, and stamped envelopes for the return of completed consent forms to the research team. A dedicated, monitored, study email address and telephone line were also created, enabling MCH nurses to contact the study team as required.

© 2022 Barbaro J et al. *JAMA Network Open.*

SACS-R and SACS-PR Interrater Reliability
To determine reliability of MCH nurses' monitoring of the SACS-R and SACS-PR items, a second rater (a researcher trained by the first author), simultaneously and independently monitored and co-coded the SACS-R and SACS-PR items with MCH nurses during routine consultations. Co-coding occurred for one child with each of 26 MCH nurses (21% of participating MCH nurses), randomly selected from all eight LGAs, with MCH nurse availability and parent/caregiver attendance (or lack thereof) at the consultation determining the number of sessions co-coded for each checklist. Four children were co-coded using the 12-month SACS-R checklist, five using the 18-month SACS-R checklist, 10 using the 24-month SACS-R checklist, and seven using the 42-month SACS-PR checklist. Inter-rater reliability was determined using percentage agreement. This method was selected as it is directly interpretable, and the raters were unlikely to "guess" their ratings due to their robust training in the SACS-R and SACS-PR tools.[8] Overall interrater agreement was ≥0.90 for all but two individual SACS-R and SACS-PR items (0.75 for 12-month "follows point" and 0.80 for 24-month "two-word utterances"). Agreement was ≥0.98 for each of the SACS-R and SACS-PR checklists, and the items from all four SACS-R checklists and the SACS-PR checklist combined (62 items).

Study Sample
As part of their routine health consultations with families, MCH nurses monitored 13 511 eligible children between the ages of 11 and 30 months using the SACS-R, from a total pool of 13 808 potentially eligible children. Parents/caregivers of 24 children declined to have their child monitored with the SACS-R; an additional 273 children were initially monitored but were ineligible to participate in the study due to being too old/young. Children were first assessed using either the 12-month checklist (n = 6458), 18-month checklist (n = 3890), or the 24-month checklist (n = 3163). Children were only monitored using the SACS-PR at their 42-month health check if they had *previously been monitored* with at least one of the SACS-R checklists. Where possible, children continued to be monitored using the age-appropriate SACS-R checklist (see eTable 1), with 79% (n = 10 701) of children monitored on more than one occasion.

In total, 31 708 SACS-R and SACS-PR assessments were completed by MCH nurses over the duration of the study, comprising of 6458 assessments for the 12-month age group, 7830 assessments for the 18-month age group, 9001 assessments for the 24-month age group, and 8419 assessments for the 42-month age group. See eFigure 2 for details regarding age group at first SACS-R assessment and number of children monitored with SACS-R on at least one occasion.

MedCalc was used to conduct a power analysis for the Receiver Operating Characteristic (ROC; alpha = .05, beta = .20 [1-Power], Area Under the Curve = .8 [good-excellent], a conservative ratio of negative/positive cases for autism = 99/1). This revealed that at .80 power, the minimum number of children required to be monitored with the SACS-R (with each checklist) and SACS-PR is 700.

Study Procedure
MCH Consultation Procedure
At the child's first consultation for the study, the MCH nurse created a record in the *Salesforce* database, entering the child's initials, date of birth, and sex. The software then generated a unique participant identification (ID) number for each child. The MCH nurse wrote this ID number on a study-specific sticker placed on the front of the child's "health record book", which allowed MCH nurses to quickly identify which children were participating in the study in an effort to track the participants at subsequent health consultations. The *Salesforce* electronic record allowed the MCH nurse to select the SACS-R assessment corresponding to the child's age at each visit and create a record of the outcomes for each assessment. During the routine 12-, 18-, 24-, and 42-month health consultations, MCH nurses assessed children using the age-appropriate SACS-R or SACS-PR checklist and selected the most appropriate result for each checklist item ('typical' or 'atypical') in the *Salesforce* database, automatically yielding a result of "high" or "low" likelihood for autism. See eTable 1 for the age ranges for the SACS-R/PR checklists, and eTable 2 for the items included in the SACS-R/ PR checklists,

Protocol for MCH Referrals to La Trobe University for Diagnostic Assessment
12-, 18-, and 24-month SACS-R assessments: Children who were rated by their MCH nurse as exhibiting 'atypical' behavior for at least three of the five 'key' SACS-R items were considered at "high likelihood" for autism and referred to the La Trobe University team for a free diagnostic assessment.

42-month SACS-PR consultations: Children rated as displaying 'atypical' behavior on at least three of the eight 'key' SACS-PR items were considered at "high likelihood" for autism. These children (n = 168), along with any

© 2022 Barbaro J et al. *JAMA Network Open.*

children who were considered at “low likelihood” for autism on SACS-PR, but whose parents/caregivers and/or MCH nurse had concerns (n = 28), were referred to the University team for a diagnostic assessment.

MCH nurses provided all parents/caregivers of children referred to the University team with an informed consent form and participant information sheet (which described the study and its procedure, how to withdraw from the study, and other relevant information) to provide permission for the University team to contact the family for the “gold standard” diagnostic assessment. For children referred following the 12- to 24-month SACS-R consultations, 15.6% (n = 51) of families did not provide consent for contact; a further 11.01% (n = 36) of families provided consent to be contacted to arrange the University assessment but subsequently declined the diagnostic assessment. For the children referred at the 42-month SACS-PR consultation, 25.00% (n = 49) of families did not provide consent for contact and 15.31% (n = 30) consented to contact but later declined the diagnostic assessment.

University Diagnostic Assessments
Prior to attending the “gold standard” diagnostic assessment, parents/caregivers completed a demographic questionnaire, which included questions regarding parental/caregiver country of birth and ethnicity to enable comparison of the sample to the population of greater Melbourne. Ethnicity options were defined by the parent/caregiver completing the questionnaire and were later grouped by the researchers for analyses. All diagnostic assessments were conducted in a large laboratory playroom with one clinician assessing the child while a second clinician simultaneously conducted the parental interview (see eTable 3 for details of instruments included in the assessments). Children underwent a diagnostic assessment every six months until they reached 30 months of age, with a final assessment undertaken at 42 months.

Arabic and Mandarin translators for both the child and parent/caregiver were utilized where required (Arabic: n = 5; Mandarin: n = 2). No other translation services were required as sufficient English was spoken and understood by the parents/caregivers and/or immediate family who also attended the assessment.

For assessments with children under 24 months of age, the Mullen Scales of Early Learning (MSEL)[9] and the Autism Diagnostic Observation Schedule – Toddler (ADOS-T)[10] were conducted with the child, with a developmental interview conducted with the parent/caregiver, due to the Autism Diagnostic Interview – Revised (ADI-R)[11] being suitable for children from 24 months of age. The developmental interview contained questions regarding the child’s developmental history, with questions focusing on the diagnostic criteria for autism, specifically social attention and communication development, language, play, and the presence of restricted, repetitive, and sensory behaviors and/or interests that are relevant for very young children (see Barbaro, Ridgway, and Dissanayake).[6] For the 24- and 30-month assessments, the MSEL and the appropriate module of the ADOS-T/ADOS-2[12] and ADI-R were utilized.

At the 42-month follow-up assessment, the ADOS and MSEL were used, with the ADI-R only repeated if the diagnostic outcome was not clear at the child’s previous diagnostic assessment (n = 24; 14.72%). In its place, a brief developmental interview was conducted for the remaining children (n = 138; 84.66%). The ADI-R was conducted for all children having a 42-month first-time assessment. Clinicians were not blinded to the child’s SACS-R/SACS-PR results or, where applicable, to the child’s previous diagnostic assessment data. This was due to the young age of the children and to provide the clinician with all available information to make a clinical judgment, which has consistently been found to produce the most stable diagnosis in very young children.[13-17]

All diagnostic assessment sessions were video recorded using two mounted, remotely adjustable cameras, which were angled to focus on the child. This was done to aid with scoring, coding, supervision, and diagnostic decisions. Detailed reports of the diagnostic assessment outcomes were provided to parents/caregivers. A total of 567 diagnostic assessments were conducted for 357 children (eFigure 2). eTable 5 shows children’s age at their first diagnostic assessment, with eTable 5 displaying the number of children who underwent single or multiple diagnostic assessments.

Diagnostic Assessment Tools
Autism Diagnostic Observation Schedule – Second Edition (Modules 1-3 and Toddler)
ADOS-2 modules 1, 2, and 3[12] and ADOS-2 Toddler (ADOS-T),[10] are standardized, semi-structured, play-based assessments designed to elicit behaviors relevant to an autism spectrum disorder (ASD) diagnosis through multiple activities. The ADOS-T is for use with children 12-30 months of age, with the ADOS-2 suitable from 31 months. All have excellent test-retest and inter-rater agreement, with intraclass correlation coefficients (ICCs) ≥0.80 for ADOS-2 for all but Module 1 restricted and repetitive behaviors (RRB) inter-rater (0.79) and test-retest for RRB in Module 1 and 2 (0.68 and 0.73, respectively) and ≥0.83 for ADOS-T for all but RRB total

© 2022 Barbaro J et al. *JAMA Network Open.*

on: inter-rater agreement for V21–30 algorithm = 0.74 and 12–20/NV21–30 algorithm = 0.75, and test-retest V21–30 algorithm = 0.60. They also have excellent sensitivity and specificity cut-off scores for identifying ASD versus other developmental conditions (≥0.81).[10,12,18] Calibrated severity scores (CSS) are reported in this study as they facilitate comparison across the various ADOS modules[19,20] and provide a measure of autism symptoms that is independent of age and language ability.[21] CSS scores range from 1 to 10 with higher scores indicating higher autism symptoms.

Austim Diagnostic Interview – Revised

The ADI-R[11] is a semi-structured diagnostic parental interview for autism assessing communication, reciprocal social interaction, play, and restricted, repetitive, and sensory behaviors over 93 items. With excellent test-retest and inter-rater agreement (ICCs ≥0.92), the ADI-R has excellent discriminant validity between individuals on the autism spectrum and individuals not on the spectrum for each of its domains.[22] The ADI-R toddler algorithm overall score was used for children at the 24-month diagnostic assessment.

Mullen Scales of Early Learning

The MSEL[9] is a standardized developmental assessment, norm-referenced for ages 0 to 68 months. The tool measures children's verbal (expressive and receptive) and non-verbal (gross and fine motor, visual reception) skills, in five subscales. It has excellent test-retest and inter-rater reliability for children aged ≤24 months (r ≥0.82).

Clinician Training and Reliability

All clinicians were registered psychologists, speech pathologists, medical doctors, postdoctoral research fellows, or PhD candidates who had been independently trained in the use of the ADOS-T/2, ADI-R, and MSEL. These clinicians were experienced in using these tools and had reached research reliability. As part of their training, clinicians shadowed the first author (a registered psychologist with 15 years' experience in the assessment of young children on the autism spectrum) for several assessments. Following this, the first author then simultaneously and independently co-coded assessments with each clinician to ensure they maintained research reliability obtained during their independent training (>80% on all items and diagnostic algorithms). The first author ensured regular supervision of the clinicians throughout the course of the study where coding, diagnosis, and other issues were discussed; monthly ADOS coding meetings were also held at the University that clinicians could attend.

Diagnostic Criteria

Information attained from children's developmental history, previous health records/reports, and the administration of the ADOS-T/ADOS-2, ADI-R, developmental interview, and MSEL, were used to make a clinical decision based on the Diagnostic and Statistical Manual of Mental Disorders (5th edition)[23] diagnostic criteria for ASD.

Diagnostic Stability

Out of the 240 children referred and assessed between 12-24 months, only two children shifted from "developmental and/or language delay" ("DD/LD") to "autism", and four from "possible autism" to "DD/LD", from 24 month to 42 months. One child shifted from "possible autism" at 18 months to "DD/LD" at 42-months (this child did not attend an assessment at 24 months).

Statistical Analyses

Currency Conversion

Australian dollars were converted to US dollars (USD) using the mid-market exchange rate available on 1 July 2014 (the approximate mid-point of the study) of USD0.949.[24]

Participants Excluded From MCH Consultation Data

The data from 273 children were excluded from the overall cohort of potentially eligible children (n = 13 808); 176 children were outside of the eligible age range at time of recruitment and 97 were entered into the database after recruitment was completed (see Figure 1 and eFigure 1).

Missing Data

Missing Data From University Diagnostic Assessments

The following data from the diagnostic assessments completed by the University team were missing:

- 24 months: four children had a developmental interview instead of the ADI-R.

© 2022 Barbaro J et al. *JAMA Network Open.*

- 42 months (follow-up): two children were missing MSEL visual reception and fine motor scores; and one child was missing the MSEL visual reception score, which meant that their Non-verbal developmental quotient (DQ) and the Overall DQ could not be calculated.
- 42 months (first-time): – one child was missing the MSEL visual reception score (so their Non-verbal DQ and Overall DQ could not be calculated); one child was missing the ADOS-2; and one child was missing the ADI-R.

Two children who were withdrawn from the study after completing the diagnostic assessment (one child who completed the 24-month assessment and one child who completed the 42-month first-time assessment). For these children the diagnostic outcome remained known but all identifiable, clinical, and assessment data were deleted.

Given the small amount of missing data (24 months: ADI n = 4, 2.1%; 42 months (follow-up): MSEL visual reception scores, n = 3, 1.8%, MSEL fine motor scores n = 2, 1.2%, non-verbal DQ n = 3, 1.8%, overall DQ n = 3, 1.8%; 42 months (first-time): MSEL visual reception score n = 1, 0.9%, ADOS n = 1, 0.9%, ADI-R n = 1, 0.9%) and recommendation to avoid mean substitution,[25] missing data from these assessments were not replaced.

Missing Data Due to Parents/Caregivers Declining a University Diagnostic Assessment and children lost to follow-up at 42-months
Of the 523 children referred for a University diagnostic assessment, parents/caregivers of 166 children declined (31.73%), offering a range of reasons for declining the initial or follow up assessment; these included having no concerns, having too many appointments, already doing early intervention, or being under the care of a pediatrician.

- *"Known outcomes":* For 73 of these 166 (43.98%) children who were referred but did not complete a University diagnostic assessment, a diagnostic outcome was established through follow-up with parents/caregivers and MCH nurses (34 with a "high likelihood" result at 12-24 months, 35 with a "high likelihood" result at 42 months, and four with a "low likelihood" result plus parental/MCH nurse concerns at 42 months). Information regarding the name of the health professional who gave the diagnosis, and date the diagnosis was given, was also collected.
- *Multiple imputation (MI):* MI[26] was used to resolve the diagnostic outcome for the 93 children (56.02%) between 12-42 months who were referred for a diagnostic outcome but did not complete a University diagnostic assessment and for whom a diagnostic outcome from the community could not be established through follow-up with parents/caregivers and MCH nurses. MI was also used for children who did not attend their SACS-PR assessment to determine their SACS-PR outcome ("high likelihood" or "low likelihood") and their final diagnostic outcome.
- MI replaces the missing data in each replication with plausible values drawn from an imputation model and was fit using logistic regression in SPSS Statistics for Windows, version 26.0.[27] As no typically developing children had been referred amongst those who did undergo a diagnostic assessment through either the study team or the community, "typically developing" was not an available option in the model. A single model was used for all missing data based on complete values for the following covariates: LGA IRSAD, sex, number of MCH consultations attended over the study duration, total number of atypical key items and overall number of atypical items based on the child's last attended MCH consultation using the SACS-R checklist items. Imputations were run five, seven, nine, and 11 times for the different sets; imputation 11 was chosen for this study as it provided replicable results, specifically for the smaller samples to avoid a 50-50 split.[28] Additional covariates and one dependent variable were added separately, depending on the outcome needed to be derived from each set. A full explanation is as follows:
    - Phase 1 – Parents/caregivers of 53 of the 327 children with "high likelihood" for autism using the SACS-R between 12-24 months declined to attend a University assessment. MI for this set included all the above-listed covariates, as well as the child's age bracket when first at "high likelihood" and the dependent variable of the observed diagnostic outcomes of the 274 children who attended a University assessment or had a known outcome from the community (total set = 327). The MI resulted in 44 children in the autism group and 9 children in the DD/LD group.
    - Phase 2 –Parents/caregivers of 39 of the 168 children with "high likelihood" using the SACS-PR and 1 child from the 28 children with "low likelihood" on the SACS-PR with parental/MCH nurse concerns declined to attend a University assessment. MI for this set included all of the above-listed covariates, as well as the "high"/"low likelihood" status of the children using SACS-PR and the dependent variable of the observed diagnostic outcomes of

the 156 children who attended a University assessment or had a known outcome from the community (total set = 196). The MI resulted in in 19 children in the "autism" group and 20 children in the "DD/LD" group from the "high likelihood" arm and 1 child in the "autism" group from the "low likelihood" plus parental/MCH nurse concerns arm.

Phase 2 not attended – there were 4951 children who did not attend their SACS-PR assessment at their 42-month MCH consultation. MI was used to determine the outcome ("high likelihood" or "low likelihood") using SACS-PR for these children. MI for this set included all the above-listed covariates, as well as the dependent variable of the observed "high" and "low likelihood" from the 8233 children who attended their SACS-PR assessment (total set = 13 184). The frequency of the likelihood was derived from imputation 11 presenting us with the pooled data for the average number of high/low likelihood outcomes. The results of this imputation yielded 97 children at "high likelihood" and 4854 at "low likelihood" out of the missing 4951 children.

To determine the number of these children who would have been in the "autism" group, we used the results of the imputation and the proportion of the observed/known outcomes data of children at "high" and "low likelihood" on the SACS-PR to estimate the number of children who would have been allocated to the "autism" group. This resulted in 63 children being allocated to the "autism" group: 57 in the "high likelihood" arm and 6 in the "low likelihood" arm. Below is an explanation of how we calculated these figures.

- Imputed "high likelihood" SACS-PR result:
  - There were 168 children with a "high likelihood" result using SACS-PR. The total number of attended + known data is 129 (94 attended + 35 known).
  - There were 76 children in the "autism" group of the 129 children (61 attended + 15 known).
  - There were 97 children with an imputed "high likelihood" SACS-PR outcome in the missing data of 4951 children.
  - The proportion of children allocated to the "autism" group out of the 97 children is: (97 x 76) /129 = 57.
- Imputed "low likelihood" SACS-PR result:
  - There were 8065 children with "low likelihood" result using SACS-PR. One child had a diagnosis imputed and not observed/known, 8065 – 1 = 8,064 children.
  - There were 10 children (8 attended + 2 known) with a "low likelihood" outcome on the SACS-PR in the "autism" group.
  - There were 4854 children with an imputed "low likelihood" SACS-PR outcome in the missing data of 4951 children.

The proportion of children allocated to the "autism" group out of the 4854 children is: (4854 x 10) / 8064 = 6.

Chi square tests and independent samples t-tests were used to identify potential differences between the children identified at "high likelihood" for autism using the SACS-R/SACS-PR who completed a University diagnostic assessment and those who did not due to their parents declining the assessment. In cases where chi square tests were run, all expected cell frequencies were >10 for 2x2 tables and >5 for all other tables. Effect size was evaluated using Cramer's V (φc). In instances where independent samples t-tests were run, the effect size was calculated using Omega Squared (ω^2). When comparing age identified at "high likelihood" for the 42-month cohort, the assumption of homogeneity of variances was violated and the p-value was determined using equal variance not assumed. For the 12- to 24-month cohort, results indicated no significant differences between the 'attended' and 'declined' groups in relation to child sex ($P = .84$) and LGA ($P = .21$) with small to moderate effect sizes (φc = -0.02 and 0.17, respectively). However, a significant difference was found between the groups for the child's age when they were first identified at "high likelihood" for autism on the SACS-R ($P = .03$) with a small effect size ($\varphi c = 0.01$), indicating that children whose parents declined a University diagnostic assessment were more likely to be younger when first identified as "high likelihood" on the SACS-R. For the 42-month cohort, there were no significant differences between the 'attended' and 'declined' groups on sex ($P = .41$) and LGA ($P = .09$), with small to large effect sizes (φc = 0.09 and 0.27, respectively). A significant difference was found between the groups for the child's age when they were first identified at "high likelihood" for autism on the SACS-PR ($P = .03$) with a small effect size ($\omega^2 = .02$), indicating children whose parents declined a University diagnostic assessment were more likely to be older when identified at "high likelihood" on the SACS-PR.

© 2022 Barbaro J et al. *JAMA Network Open.*

Assumption Testing
The level of measurement assumption and the assumption of independence of observations was met. Outliers were detected across multiple variables, as assessed by inspection of boxplots. Analyses were run to compare the results with and without outliers, with similar results; as the outliers were not found to influence the results, these were not removed. Normality was assessed using visual inspection of histograms and Shapiro-Wilks Tests of Normality. The assumption of normality was violated for:

- Chronological age at 24-month, 42-month (follow-up) and 42-month (first time) assessments,
- Non-verbal DQ at 18-month and 42-month (first time) assessments,
- Verbal DQ at 18-month, 24-month and 42-month (follow up) assessments,
- Overall DQ at 24-month and 42-month (follow-up) assessments,
- ADI-R Toddler overall total score at 24-month assessments,
- ADI-R Communication (Verbal) at 42-month (first-time) assessments,
- ADI-R RRB at 42-month (follow-up) and 42-month (first-time) assessments, and
- ADOS-2 CSS at 12-month, 18-month, 24-month, 42-month (follow-up), and 42-month (first-time) assessments.

In cases where normality assumptions were violated, Mann Whitney U tests were used.

Diagnostic Accuracy
Calculation of specificity, sensitivity, PPV, and NPV (see eTables 6 and 7) were calculated via the following formulas:

		**Autism**		
		**Autism present**	**Autism absent**	
**SACS-R/PR outcome**	**SACS-R/PR positive (“high likelihood”)**	**True positive (TP)**	**False positive (FP)**	**PPV** = TP / (TP + FP)
	**SACS-R/PR negative (“low likelihood”)**	**False negative (FN)**	**True negative (TN)**	**NPV** = TN / (TN + FN)
		**Sensitivity** = TP / (TP + FN)	**Specificity** = TN / (FP + TN)	

Where:

- **True positive (TP):** Children on the autism spectrum *correctly* identified at “high likelihood” for autism.
- **False positive (FP):** Children not on the autism spectrum *incorrectly* identified at “high likelihood” for autism.
- **True negative (TN):** Children not on the autism spectrum *correctly* identified at “low likelihood” for autism.
- **False negative (FN):** Children on the autism spectrum *incorrectly* identified at “low likelihood” for autism.

eResults. Supplementary Results.

Sample Characteristics: Demographics, Autism Symptoms, and Developmental Scores
The summary of clinical assessment scores by age group and final diagnostic status are presented in eTable 4. Given the large amount of data generated in this study, only significant findings from eTable 4 are reported here.

Comparisons between the autism and DD/LD groups using independent t-tests revealed statistically significant differences in chronological age at 12- and 18-month assessments and the 42-month (first-time) assessments, with small to large effect sizes ($\omega^2 = 0.15$, $\omega^2 = 0.08$, and $r = .21$, respectively). Correlation analyses were run to determine the strength of the relationship between chronological age and the other dependent variables tested. Based on alpha <.05 none of the dependent variables were significantly related to age thus it was not controlled for in group comparisons.

At the 12-month assessment, the autism group had significantly higher ADOS-2 CSS, with a small effect size ($r = 0.18$).

At the 18-month assessment, the DD/LD group had significantly higher verbal DQ and overall DQ, with small to medium effect sizes ($r = 0.33$ and $\omega^2 = 0.04$, respectively). The autism group had significantly higher ADOS-2 CSS, with large effect size ($\omega^2 = -0.54$).

© 2022 Barbaro J et al. *JAMA Network Open.*

Similarly, at the 24-month assessment the DD/LD group had significantly higher verbal DQ and overall DQ, with small effect sizes (r = 0.26 and 0.23 respectively). The autism group had significantly higher ADOS-2 CSS in addition to significantly higher ADI-R Toddler overall total scores; effect sizes were large (r = 0.58 and 0.51, respectively).

At the 42-month (follow-up) assessments the DD/LD group had significantly higher non-verbal DQ, verbal DQ, and overall DQ; effect sizes were small (ω^2 = 0.03, r = 0.21, and r = 0.20, respectively). The autism group had significantly higher ADOS CSS, with a large effect size (r = 0.63) and higher ADI-R social interaction and ADI-R communication (verbal) domains; effect sizes were large (ω^2 = 0.12 and ω^2 = 0.23, respectively).

For 42-months (first-time) assessments, group differences were significant for every variable tested. The DD/LD group had significantly higher non-verbal DQ, verbal DQ, and overall DQ, with small to medium effect sizes (r = 0.40, ω^2 = 0.06, and r = 0.13, respectively). The autism group had significantly higher ADOS-2 CSS (r = -.64) and higher ADI-R scores on all domains, with a range of medium to large effect sizes (r = -.53, ω^2 = 0.58, r = -.44 and 0.35, respectively).

© 2022 Barbaro J et al. *JAMA Network Open.*

eFigure 1. Detailed STARD Study Flow Diagram.

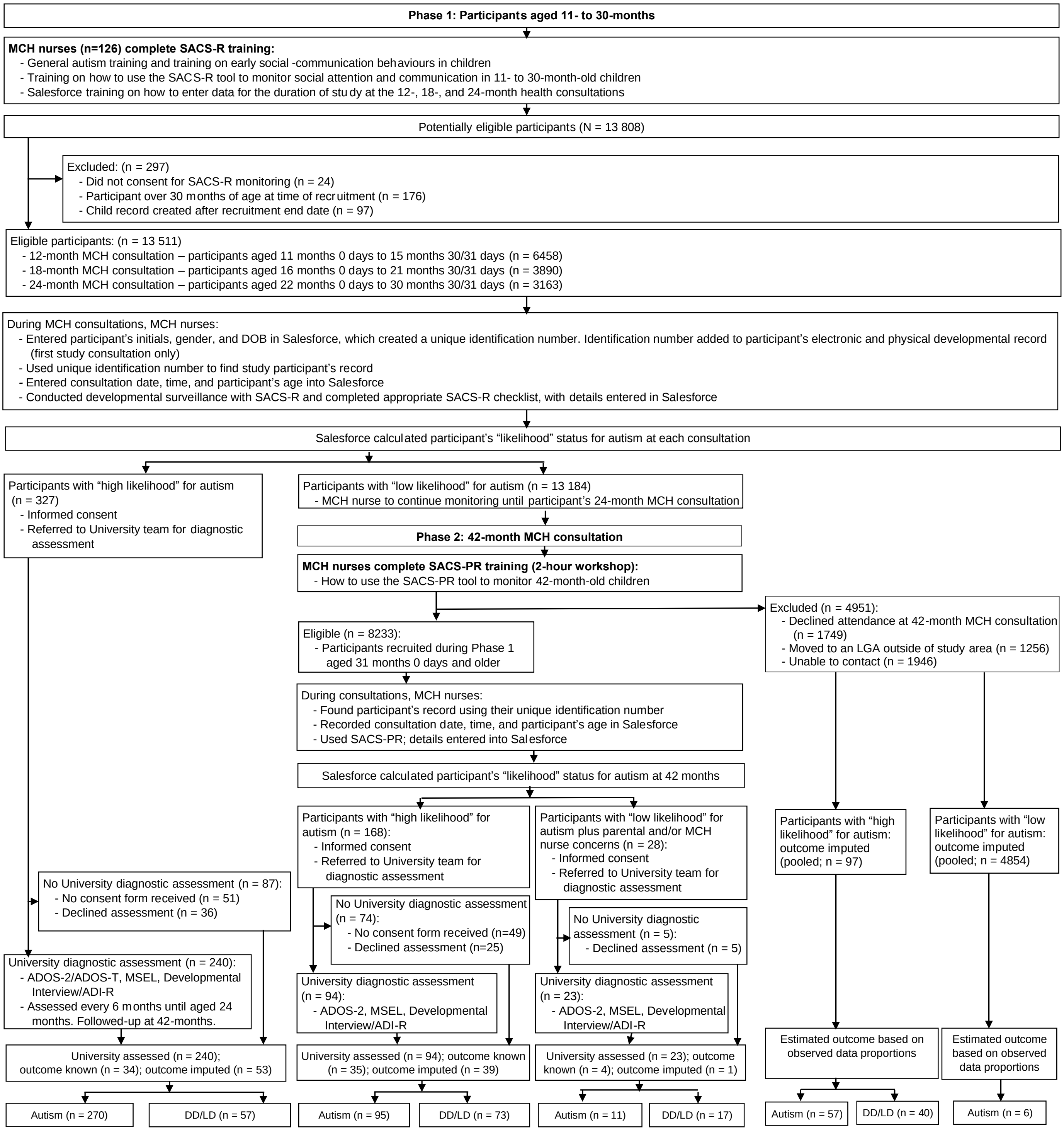

Abbreviations: ADI-R, Autism Diagnostic Interview – Revised; ADOS-2/Toddler, Autism Diagnostic Observation Schedule Second Edition/Toddler Module; DD/LD, developmental delay and/or language delay; MCH, Maternal and Child Health; MSEL, Mullen Scales of Early Learning; SACS-PR, Social Attention and Communication Surveillance-Preschool; SACS-R, Social Attention and Communication Surveillance-Revised.

© 2022 Barbaro J et al. *JAMA Network Open.*

eTable 1. Age ranges for the SACS-R and SACS-PR checklists.

	Minimum age	Maximum age
**SACS-R checklists**		
12-Months	11 months 0 days	15 months 30/31 days
18-Months	16 months 0 days	21 months 30/31 days
24-Months	22 months 0 days	30 months 30/31 days
**SACS-PR checklist**		
42-Months	31 months 0 days	60 months 30/31 days

Abbreviations: SACS-PR, Social Attention and Communication Surveillance-Preschool; SACS-R, Social Attention and Communication Surveillance-Revised.

© 2022 Barbaro J et al. *JAMA Network Open.*

eTable 2. Behaviors Monitored by SACS-R at 12-, 18-, and 24-Months, and SACS-PR at 42-Months, Including 'Key' Items.

Behavior	Age at which behavior is monitored			
	SACS-R			SACS-PR
	12-months	18-months	24-months	42-months
Pointing	✓ K	✓ K	✓ K	✓ K
Eye contact	✓ K	✓ K	✓ K	✓ K
Waving "bye, bye"	✓ K	✓ K	✓ K	
Response to name	✓ K	✓	✓	✓
Imitation	✓ K	✓	✓	
Social communication (showing)		✓ K	✓ K	✓ K
Pretend play		✓ K	✓ K	✓ K
Follows point	✓	✓	✓	✓
Social smile	✓	✓	✓	✓
Conversational babble	✓			
Says 1-3 clear words	✓			
Attending to sounds	✓			
Understands/obeys simple instructions	✓	✓		
Uses 5-10 words		✓		
Understands words		✓		
Points to facial features		✓		
Loss of skills		✓	✓	✓
Uses 20-50 words			✓	
2-word utterances			✓	
Interest in other children			✓	
Use/understanding of language			✓	
Parallel play			✓	
Follows 2 unrelated commands				✓ K
Odd/unusual speech				✓ K
Sensory behaviors/interests				✓ K
Reciprocal social interaction				✓ K
Gestures				✓
Sharing interest				✓
Uses 5-6 word sentences				✓
Conversation				✓
Hand as tool				✓
Immediate echolalia				✓

© 2022 Barbaro J et al. *JAMA Network Open.*

Pronoun reversals				✓
Repetitive speech				✓
Motor stereotypies				✓
Repetitive/restricted behaviors and interests				✓

Abbreviations: K, Key item; SACS-PR, Social Attention and Communication Surveillance-Preschool; SACS-R, Social Attention and Communication Surveillance-Revised.

© 2022 Barbaro J et al. *JAMA Network Open.*

eTable 3. Tools Administered During University Diagnostic Assessments Undertaken at Each Age Group.

	Age group at assessment			
	12-months	18-months	24-months	42-months
Autism Diagnostic Observation Schedule – Second Edition, Modules: Toddler, 1, 2, or 3 (dependent on age or language skills)	✓	✓	✓	✓
Mullen Scales of Early Learning	✓	✓	✓	✓
Developmental Interview	✓	✓		✓
Autism Diagnostic Interview-Revised			✓	✓
Demographic Questionnaire	✓	✓	✓	✓

© 2022 Barbaro J et al. *JAMA Network Open.*

eFigure 2. Phase 1 Cohort Recruitment and Monitoring by Age Group.

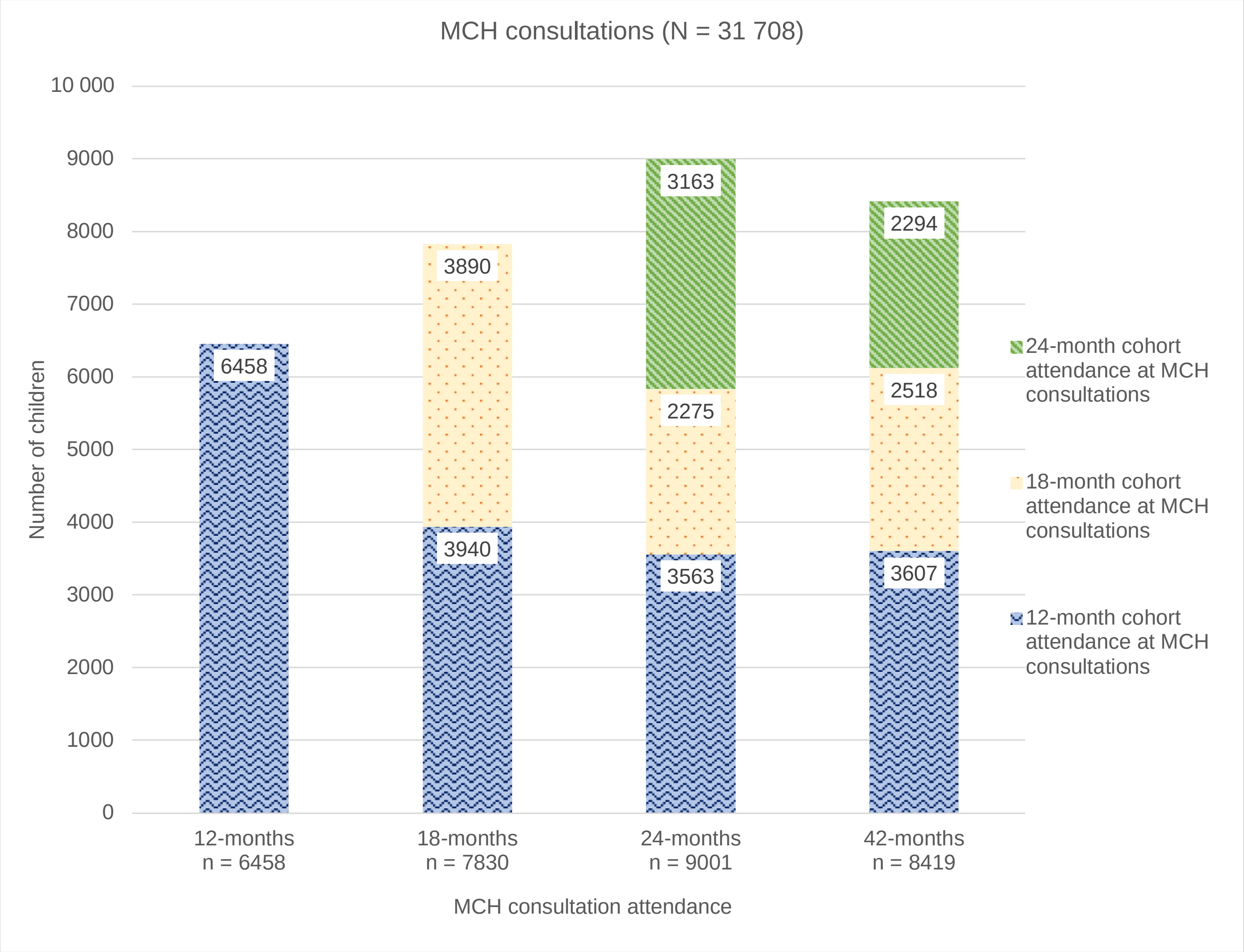

Abbreviation: MCH, Maternal and Child Health.

© 2022 Barbaro J et al. *JAMA Network Open.*

eTable 4. Clinical Assessment Scores for All University Assessments, Grouped by Age at Assessment and Outcome.

	Autism		DD/LD			
	Mean (SD)	Median (IQR)	Mean (SD)	Median (IQR)	*P* value	Effect size
**12-month assessment (N = 23)**	n = 16		n = 7			
Chronological age, months	14.0 (0.9)	13.9 (13.4-14.9)	14.8 (0.4)	14.9 (14.6-15.2)	.04	.15 (ω^2)
Non-verbal DQ	103.3 (17.3)	103.3 (96.7-110.6)	108.5 (13.0)	113.3 (96.4-117.9)	.49	-.02(ω^2)[a]
Verbal DQ	76.8 (13.9)	78.5 (64.8-84.6)	84.3 (4.4)	83.3 (82.1-89.2)	.07	.11 (ω^2)
Overall DQ	90.1 (12.8)	87.5 (81.0-100.4)	96.4 (7.5)	95.0 (89.3-103.6)	.24	.02 (ω^2)
ADOS-T CSS	5.8 (1.9)	5.0 (4.3-7.0)	2.9 (0.9)	3.0 (3.0-3.0)	.001	.18 (r)
Sex, n (%)						
Female	1 (6.25)		1 (14.29)			
Male	15 (93.75)		6 (85.71)			
Sex ratio, female to male	1:15		1:6			
**18-month assessment (N = 78)**	n = 63		n = 15			
Chronological age, months	19.7 (1.3)	19.9 (18.7-20.5)	20.7 (0.8)	20.5 (20.3-21.5)	.008	.08 (ω^2)
Non-verbal DQ	85.8 (16.7)	90.0 (75.0-97.1)	87.2 (16.1)	88.1 (76.2-100.0)	.91	.01 (r)
Verbal DQ	63.3 (22.2)	58.3 (47.5-73.5)	80.1 (19.2)	80.0 61.9-95.2)	.004	.33 (r)
Overall DQ	74.5 (16.4)	75.0 (67.1-82.9)	83.6 (15.1)	84.5 (71.3-95.2)	.05	.04 (ω^2)
ADOS-T CSS	7.0 (2.2)	7.0 (6.0-9.0)	3.2 (2.0)	3.0 (2.0-4.0)	<.001	-.54 (ω^2)
Sex, n (%)						
Female	13 (20.63)		6 (40.00)			
Male	50 (79.37)		9 (60.00)			
Sex ratio, female to male	1:3.8		1:1.5			
**24-month assessment (N = 185)**	n = 151[b]		n = 34			
Chronological age, months	27.2 (2.7)	26.7 (25.4-28.6)	27.0 (2.4)	26.5 (25.3-27.7)	.67	.03 (r)
Non-verbal DQ	81.8 (18.4)	80.0 (70.5-92.3)	87.9 (14.1)	86.6 (79.6-98.9)	.07	.01 (ω^2)
Verbal DQ	65.2 (26.4)	61.3 (46.2-83.8)	81.5 (22.1)	78.1 (66.5-94.2)	<.001	.26 (r)
Overall DQ	73.5 (20.8)	70.2 (60.5-86.7)	84.7 (16.3)	82.5 (73.3-96.0)	.002	.23 (r)
ADOS-2 CSS	7.1 (2.3)	7.0 (5.0-9.0)	2.9 (1.2)	3.0 (2.0-4.0)	<.001	.58 (r)
ADI-R Toddler algorithm overall total score[b]	14.4 (5.9)	14.0 (10.0-19.0)	6.0 (4.3)	4.5 (3.0-9.3)	<.001	.51 (r)
Sex, n (%)						
Female	29 (19.21)		7 (20.59)			
Male	122 (80.79))		27 (79.41)			
Sex ratio, female to male	1:4.2		1:3.9			
**42-month (follow-up) assessment (N = 163)**	n = 133		n = 30			
Chronological age, months	44.3 (4.7)	44.1 (42.3-46.3)	44.2 (2.9)	43.5 (42.5-45.3)	.74	.03 (r)
Non-verbal DQ[b]	80.0 (25.7)	79.5 (61.5-98.8)	91.6 (18.2)	88.2 (79.1-109.5)	.02	.03 (ω^2)

© 2022 Barbaro J et al. *JAMA Network Open.*

	Autism		DD/LD			
	**Mean (SD)**	**Median (IQR)**	**Mean (SD)**	**Median (IQR)**	***P* value**	**Effect size**
Verbal DQ	76.0 (33.1)	80.4 (49.4-97.6)	92.8 (19.9)	91.8 (78.4-107.8)	.009	.21 (r)
Overall DQ[b]	78.0 (28.7)	81.1 (51.7-98.7)	92.2 (17.8)	93.4 (75.8-104.9)	.01	.20 (r)
ADOS-2 CSS	6.7 (1.7)	6.0 (6.0-8.0)	2.7 (1.5)	3.0 (1.0-4.0)	<.001	.62 (r)
ADI-R social interaction	11.0 (5.8)	11.0 (6.8-15.3)	5.9 (4.4)	5.0 (2.0-9.0)	.003	.12 (ω^2)
ADI-R communication (non-verbal)[c]	8.2 (3.8)	9.0 (6.0-11.0)	10.0 (-)	10 (-)	.65	-.05 (ω^2)[a]
ADI-R communication (verbal)[c]	9.6 (4.1)	9.0 (6.0-13.0)	4.5 (3.8)	3.5 (1.0-7.3)	<.001	.23 (ω^2)
ADI-R RRB[d]	4.9 (2.6)	5.0 (3.0-6.0)	3.7 (2.8)	3.0 (2.0-7.0)	.18	.17 (r)
Sex, n (%)						
Female	25 (18.80)		9 (30.00)			
Male	108 (81.20)		21 (70.00)			
Sex ratio, female to male	1:4.3		1:2.3			
**42-month (first-time) assessment (N = 117)**	n = 68[b]		n = 48			
Chronological age, months	45.8 (5.8)	46.0 (43.2-49.0)	48.0 (4.5)	47.1 (44.9-51.3)	.03	.21 (r)
Non-verbal DQ[b]	88.0 (20.4)	90.7 (71.2-104.3)	104.1 (16.1)	103.3 (94.0-114.4)	<.001	.40 (r)
Verbal DQ	90.2 (23.4)	93.0 (73.0-107.4)	102.5 (19.1)	102.3 (102.3-114.5)	.003	.06 (ω^2)
Overall DQ[b]	89.1(20.6)	90.0 (75.1-103.7)	103.4 (15.6)	104.1 (104.1-111.4)	<.001	.13 (ω^2)
ADOS-2 CSS	6.8 (1.7)	7.0 (6.0-8.0)	3.6 (2.1)	3.0 (2.0-5.0)	<.001	-.64. (r)[a]
ADI-R social interaction	10.6 (5.1)	10.0 (7.0-13.0)	5.5 (3.0)	5.0 (4.0-7.6)	<.001	-.53 (r)[a]
ADI-R communication (non-verbal)[e]	9.5 (2.5)	9.5 (7.5-12.0)	2.5 (2.1)	2.5 (1.0-)[f]	.01	.58 (ω^2)
ADI-R communication (verbal)[e]	9.9 (4.9)	9.0 (6.0-14.0)	5.7 (3.8)	5.0 (3.0-8.0)	<.001	-.44. (r)[a]
ADI-R RRB	5.3 (2.6)	5.0 (3.0-7.0)	2.5 (1.2)	3.0 (2.0-5.0)	<.001	-.35. (r)[a]
Sex, n (%)						
Female	16 (23.53)		7 (14.58)			
Male	52 (76.47)		41 (85.42)			
Sex ratio, female to male	1:3.3		1:5.9			

Abbreviations: ADI-R communication (non-verbal), Autism Diagnostic Interview – Revised: qualitative abnormalities in communication (non-verbal) algorithm total; ADI-R communication (verbal), Autism Diagnostic Interview – Revised: qualitative abnormalities in communication (verbal) algorithm total; ADI-R RRB, Autism Diagnostic Interview – Revised: restricted, repetitive and stereotyped patterns of behavior algorithm total; ADI-R social interaction, Autism Diagnostic Interview – Revised: qualitative abnormalities in reciprocal social interaction algorithm total; ADI-R Toddler algorithm overall total score, Autism Diagnostic Interview – Revised Toddler algorithm overall total score; ADOS-2 CSS, Autism Diagnostic Observation Schedule – Second Edition calibrated severity score; ADOS-T CSS, Autism Diagnostic Observation Schedule – Toddler Module calibrated severity score; DD/LD, developmental and/or language delay; DQ, Developmental Quotient.

[a] Negative effect sizes were obtained and were reported as is, as recommended by Okada.[51]

[b] Missing data/children withdrawn; please see eMethods for further details

[c] ADI-R Communication (non-verbal) n = 13 (Autism n = 12; DD/LD n = 1) and ADI-R Communication (verbal) n = 47 (Autism n = 33; DD/LD n = 14)

[d] ADI-R n = 60 (Autism n = 45; DD/LD n = 15)

[e] ADI-R Communication (non-verbal) n = 8 (Autism n = 6; DD/LD n = 2) and ADI-R Communication (verbal) n=107 (Autism n = 61; DD/LD n = 46)

[f] No upper IQR limit due to number of participants in this group (n = 2)

© 2022 Barbaro J et al. *JAMA Network Open.*

eTable 5. Children Who Underwent Diagnostic Assessments One or More Times.

	First diagnostic assessment (age group)	Break-down of diagnostic assessments	Number of diagnostic assessments at each age group				Total number of diagnostic assessments
			12 months	18 months	24 months	42 months	
**Phase 1**<br><br>Diagnostic assessments of children at **"high likelihood" of autism on SACS-R**<br>- N = 240	12 months; n = 23	6 children assessed at 12 months only	6	-	-	-	6
		2 children assessed at 12, 18, and 24 months	2	2	2	-	6
		5 children assessed at 12, 18, 24, and 42 months	5	5	5	5	20
		3 children assessed at 12, 18, and 42 months	3	3	-	3	9
		1 child assessed at 12 and 24 months	1	-	1	-	2
		6 children assessed at 12, 24, and 42 months	6	-	6	6	18
	18 months; n = 68	15 children assessed at 18 months only	-	15	-	-	15
		10 children assessed at 18 and 24 months	-	10	10	-	20
		37 children assessed at 18, 24, and 42 months	-	37	37	37	111
		6 children assessed at 18 and 42 months	-	6	-	6	12
	24 months; n = 125	43 children assessed at 24 months only	-	-	43	-	43
		82 children assessed at 24 and 42 months	-	-	82	82	164
	42 months; n = 24	24 children assessed at 42 months only	-	-	-	24	24
**Phase 2**<br><br>Diagnostic assessments of children at **"high likelihood" of autism on SACS-PR** or due to parental and/or MCHN concerns at 42 months<br>- N = 117	42 months; n = 117	117 children assessed at 42 months	-	-	-	117	117
No. of diagnostic assessments conducted at each age group			23	78	186	280	567

Abbreviations: SACS-PR, Social Attention and Communication Surveillance-Preschool; SACS-R, Social Attention and Communication Surveillance-Revised.

© 2022 Barbaro J et al. *JAMA Network Open.*

eTable 6. Results of Analysis for SACS-R Between 12-24 Months.

**Cross Tabulation**			
	**Final Diagnostic Outcome**		
**SACS-R Result**	**Autism**	**Non-Autism**	**Total**
“High likelihood” for autism	270 True Positive	57 False Positive	327
“Low likelihood” for autism	169 False Negative	13 015 True Negative	13 184
**Total**	439	13 072	13 511

Abbreviations: CI, confidence interval; NPV, negative predictive value; PPV, positive predictive value; SACS-R, Social Attention and Communication Surveillance-Revised.

© 2022 Barbaro J et al. *JAMA Network Open.*

eTable 7. Results of Analysis for SACS-R+PR Between 12-42 Months.

Cross Tabulation			
	**Final Diagnostic Outcome**		
**SACS-R+PR Result**	**Autism**	**Non-Autism**	**Total**
"High likelihood" for autism	422 True Positive	170 False Positive	592
"Low likelihood" for autism	17 False Negative	12 902 True Negative	12 919
**Total**	439	13 072	13 511

Abbreviations: CI, confidence interval; NPV, negative predictive value; PPV, positive predictive value; SACS-R+PR, Social Attention and Communication Surveillance-Revised+Preschool

© 2022 Barbaro J et al. *JAMA Network Open.*

References

1. Department of Education and Training. *Memorandum of Understanding between Department of Education and Training and Municipal Association of Victoria in relation to the Maternal and Child Health Service 2017 – 2020.* Melbourne, Victoria: Victorian Government; 2017.
2. Department of Early Education and Child Development. *Maternal and Child Health Service: Practice Guidelines 2009.* Melbourne: Victorian Government; 2009.
3. Department of Education and Training. Maternal and child health services annual report 2016-2017 statewide. In. Melbourne: Victorian Government; 2017.
4. Department of health and Human Services. *Maternal and Child Health Service guidelines.* Melbourne, Australia: Victorian Government; 2019.
5. Australian Bureau of Statistics. *Census of Population and Housing: Socio-Economic Indexes for Areas (SEIFA), Australia, 2016.* 2016.
6. Barbaro J, Ridgway L, Dissanayake C. Developmental surveillance of infants and toddlers by maternal and child health nurses in an Australian community-based setting: promoting the early identification of autism spectrum disorders. *J Pediatr Nurs.* 2011;26(4):334-347.
7. Manna M. A Cloud-Based Encryption for Document Storage Using Salesforce.com. *Journal of Engineering and Applied Sciences.* 2018;13:2382-2387.
8. McHugh ML. Interrater reliability: the kappa statistic. *Biochemia medica.* 2012;22(3):276-282.
9. Mullen EM. *Mullen Scales of Early Learning.* Circle Pines, NM: American Guidance Service; 1995.
10. Lord C, Luyster R, Gotham K, Guthrie W. *Autism Diagnostic Observation Schedule, Second Edition (ADOS-2) Manual (Part II): Toddler Module.* Torrance, CA: Western Psychological Services; 2012.
11. Rutter M, Le Couteur A, Lord C. *Autism Diagnostic Interview - Revised (ADI-R) Manual.* Los Angeles: Western Psychological Services; 2003.
12. Lord C, Rutter M, DiLavore P, Risi S, Gotham K, Bishop S. *Autism Diagnostic Observation Schedule, Second Edition.* Torrence, CA: Western Psychological Services; 2012.
13. Barbaro J, Dissanayake C. Autism Spectrum Disorders in Infancy and Toddlerhood: A Review of the Evidence on Early Signs, Early Identification Tools, and Early Diagnosis. *J Dev Behav Pediatr.* 2009;30(5):447-459.
14. de Bildt A, Sytema S, Ketelaars C, et al. Interrelationship between Autism Diagnostic Observation Schedule-Generic (ADOS-G), Autism Diagnostic Interview-Revised (ADI-R), and the Diagnostic and Statistical Manual of Mental Disorders (DSM-IV-TR) classification in children and adolescents with mental retardation. *J Autism Dev Disord.* 2004;34(2):129-137.
15. Le Couteur A, Haden G, Hammal D, McConachie H. Diagnosing Autism Spectrum Disorders in Pre-school Children Using Two Standardised Assessment Instruments: The ADI-R and the ADOS. *J Autism Dev Disord.* 2008;38(2):362-372.
16. Pierce K, Gazestani VH, Bacon E, et al. Evaluation of the Diagnostic Stability of the Early Autism Spectrum Disorder Phenotype in the General Population Starting at 12 Months. *JAMA Pediatr.* 2019;173(6):578-587.
17. Stone WL, Lee EB, Ashford L, et al. Can autism be diagnosed accurately in children under 3 years? *J Child Psychol Psychiatry.* 1999;40(2):219-226.
18. Luyster R, Gotham K, Guthrie W, et al. The Autism Diagnostic Observation Schedule—Toddler Module: A New Module of a Standardized Diagnostic Measure for Autism Spectrum Disorders. *J Autism Dev Disord.* 2009;39(9):1305-1320.
19. Esler AN, Bal VH, Guthrie W, Wetherby A, Weismer SE, Lord C. The Autism Diagnostic Observation Schedule, Toddler Module: Standardized Severity Scores. *J Autism Dev Disord.* 2015;45(9):2704-2720.
20. Gotham K, Pickles A, Lord C. Standardizing ADOS Scores for a Measure of Severity in Autism Spectrum Disorders. *J Autism Dev Disord.* 2009;39(5):693-705.
21. Shumway S, Farmer C, Thurm A, Joseph L, Black D, Golden C. The ADOS Calibrated Severity Score: Relationship to Phenotypic Variables and Stability over Time. *Autism Res.* 2012;5(4):267-276.
22. Lord C, Rutter M, Le Couteur A. Autism Diagnostic Interview-Revised: a revised version of a diagnostic interview for caregivers of individuals with possible pervasive developmental disorders. *J Autism Dev Disord.* 1994;24(5):659-685.
23. American Psychiatric Association. *Diagnostic and Statistical Manual of Mental Disorders.* 5th ed. Arlington, VA: Author; 2013.
24. xe.com. Currency Table: AUD — Australian Dollar. https://www.xe.com/currencytables/?from=AUD&date=2014-07-01#table-section. Accessed 19/11/2021.
25. Kang H. The prevention and handling of the missing data. *Korean J Anesthesiol.* 2013;64(5):402-406.

26. Rubin DB. Inference and Missing Data. *Biometrika.* 1976;63(3):581-592.
27. *IBM SPSS Statistics for Windows* [computer program]. Version 26. Armonk, NY: IBM Corp; 2019.
28. Rubin DB. *Multiple imputation for nonresponse in surveys.* New York: Wiley; 1987.

© 2022 Barbaro J et al. *JAMA Network Open.*
